# Metagenomic profiles of the early life microbiome of Indonesian inpatient neonates and their influence on clinical characteristics

**DOI:** 10.1038/s41598-022-13496-4

**Published:** 2022-06-07

**Authors:** Radhian Amandito, Amarila Malik, Rinawati Rohsiswatmo

**Affiliations:** 1 Division of Perinatology, Department of Child Health, Cipto Mangunkusumo Hospital-Faculty of Medicine, Universitas Indonesia, Jl. Pangeran Diponegoro No. 71, Central Jakarta, Jakarta 10430 Indonesia; 2 Division of Pharmaceutical Microbiology and Biotechnology, Faculty of Pharmacy, Universitas Indonesia, UI Depok Campus, Depok, 16424 Indonesia

**Keywords:** Microbiota, Paediatric research

## Abstract

Determining the initial normal neonatal gut microbiome is challenging. The debate regarding the sterile fetal environment is still ongoing. Therefore, studying and comparing normal and dysbiotic microbiomes requires the elucidation of both the fetal and infant microbiomes. Factors influencing the normal microbiome also include regional and genetic factors specific to different countries. Determining the normal microbiome population in our center and their association with the clinical conditions of infants is helpful as a tool for both the prevention and treatment of related diseases during neonatal care. Here, we employed metagenomic sequencing to characterize meconium and the subsequent early-life gut microbiome of preterm neonates in Jakarta, Indonesia. Microbiome diversity and complexity was higher in the meconium and on day 4 than on day 7. At the genus level, the most abundant genus overall was unidentified *Enterobacteriaceae*, with meconium samples dominated by *Ureaplasma*, day 4 fecal samples dominated by *Staphylococcus*, and day 7 samples dominated by *Clostridiales*, while at the phylum level the most abundant was *Proteobacteria* and *Firmicutes.* Perinatal factors of PROM and mother’s diet influenced the meconium microbiome, while day 4 and day 7 microbiome was associated with bacteremia and early administration of antibiotics. One of our sample sets was derived from triplets, and they had varying diversity despite being triplets. These data are valuable for understanding the formation of a healthy microbiome specific to neonates and devising a strategy to improve both the gut health and related clinical outcomes of the neonate.

## Introduction

Understanding the fetal microbiome and the interaction between diseases of newborns and their subsequent gut microbiome is promising as both a diagnostic and therapeutic instrument^1,2^. To date, knowledge regarding this field remains insufficient^3^. For many years, the fetus and the intrauterine environment have been known to be sterile, with the earliest microbial exposure and colonization occurring during delivery, promptly resulting in acquisition of maternal and environmental bacteria^4^. The microbiome initially has low diversity and low complexity and eventually develops and reaches a mature and enduring state after 2–3 years of age^1,4,5^.

There are differences in the colonization and development of the gut microbiome between preterm and term infants^6^. Preterm infants exhibit many factors that affect colonization, including antibiotic therapy for the mother or infant, cesarean section, early separation from parents, delayed enteral feeding, invasive medical procedures, or prolonged stay in the neonatal intensive care unit (NICU)^6–8^. Compared with term infants, preterm infants also have a reduced number of bacterial species in their fecal microbiome, which contributes to the difficulty of detecting the bacteria when using culture-based methods^9,10^. Establishment of obligate anaerobes in preterm infants’ fecal microbiome is delayed, and these organisms persist for several weeks at high levels. This abnormal colonization in preterm infants during their first weeks of life may affect the maturation of the gut barrier as well as its nutritional and immunological functions during that time or later in life^2,11–13^.

Specific types of bacteria and their products may produce a dysfunctional gut-brain axis (GBA) via the production of aberrant metabolites, and their immune effects impair fetal brain development, immune development, especially the early response to neonatal sepsis, and growth, subsequently leading to life-long outcomes^2,3,11^. In addition, perinatal factors, including maternal diet, mode of delivery, and maternal stress, have been documented to influence the infant gut microbiome^14^. Thus, understanding the importance of the maternal condition and the subsequent consequences of impaired condition for the neonate will emphasize the need for preventative measures for pregnant women, as specific bacteria stimulate the release of proinflammatory cytokines, prostaglandins, and matrix metalloproteinases, which leads to preterm birth^4,12^. This information may be helpful to guide neonatologists when receiving a new neonate and when treating them in the NICU regarding what to expect and what treatments may be necessary.

We studied the meconium and fecal samples of preterm infants from a referral center hospital in Jakarta, Indonesia, applying next-generation sequencing (NGS); prior to this study, we applied a culture-based method to first map the microbiome of our hospital’s infants. Our aim is to characterize the early life fecal microbiome and to determine their extent of influences towards the neonates’ clinical conditions. Herein, we demonstrated the effect of clinical and environmental factors on meconium and the subsequent day 4 and day 7 fecal microbiome and their associations with clinical outcomes in the NICU.

## Materials and methods

### Study design, participants, and fecal samples

This prospective cohort observational study included eight inpatient neonates born from February to June 2020 at Cipto Mangunkusumo Hospital (CMH), Jakarta (Indonesia). Fecal samples, i.e., the meconium and the day 4 and day 7 fecal samples, were collected by CMH-trained laboratory personnel from all neonates.

We excluded neonates with signs of hemolytic anemia caused by ABO incompatibility (based on blood grouping, Coomb's test, and decreased hemoglobin levels), cephalhematoma, or major congenital malformations assessed through clinical and laboratory examinations. Subgroups were then formed for further analysis between variables based on the number of patients with specific variables.

Relevant clinical data recorded for each neonate, such as delivery mode, feeding pattern, gestational age, necrotizing enterocolitis (NEC) status (based on radiologic and clinical findings), and sepsis status (proven through positive blood culture), are detailed in Table 1.

**Table 1:** Characteristic of preterm inborn in-patient neonates in this study.

Variables	Preterm inborn-in-patient neonates (subjects)							
	CIT	JWR1	JWR2	JWR3	SRI	MIR	ADS	AJM
**Gender**								
Male (M)			M	M		M	M	
Female (F)	F	F			F			F
**Gestational age (weeks)**								
Moderate to Late Preterm	33	33	33	33		34		
Very Preterm					32		30	31
**Delivery mode**								
Caesarean Section (CS)	CS	CS	CS	CS	CS	CS	CS	
Vaginal Route (VR)								VR
**Birth weight (g)**								
Low Birth Weight (LBW)	1765		1900	1705	1305		1570	
Very Low Birth Weight (VLBW)		1130				1080		1260
**Feeding pattern**								
Breast milk (BM)	BM				BM	BM	BM	BM
Mix (Mx)		Mx	Mx	Mx				
**Antibiotics administered**								
Yes	Yes	Yes	Yes	Yes			Yes	
No					No	No		No
**Antibiotics during pregnancy**								
Yes	Yes							
No		No	No	No	No	No	No	No
**Sepsis**								
Yes								
No	No	No	No	No	No	No	No	No
**Necrotizing enterocolitis**								
Yes								
No	No	No	No	No	No	No	No	No
**Total serum bilirubin (TSB value)**	8.17		11.08		8.28	9.67		
Apgar score (A/S)	5/8	6/10	6/10	6/10	8/9	9/10	2/8	6/9
**Chorioamnionitis risk**	+	−	−	−	+	−	+	−

The meconium samples and the day 4 and day 7 fecal samples from the same inpatient neonates born at CMH were collected from diapers and put into sterile tubes, which was performed similarly throughout the study. The fecal samples were stored in a cooler box at 4 °C before being transported to Universitas Indonesia, Depok, on the same day. All samples were then kept in a refrigerator before processing for DNA extraction.

All experimental protocols were approved by the ethics committee of the Faculty of Medicine, Universitas Indonesia. Informed consent was obtained from all subjects’ legal guardian(s).

### Preparation of DNA for next-generation sequencing

The DNA from meconium samples and fecal samples was prepared on the same day after collection and treated to obtain the optimum yield of high-quality DNA for the NGS process. This step was carried out as elaborated by Rulita et al.^15^.

Amplification of the V3–V4 region of the 16S rRNA (16S) gene using the specific primers forward 515F and reverse 806R and subsequent NGS were carried out by Novogen Co., China.

### Bioinformatic data analysis

To analyze the 16S rRNA gene sequences, bioinformatic analysis was performed by Novogen Co., China, as follows.

### Sequencing data processing

Paired-end reads were assigned to samples based on their unique barcodes and truncated by trimming the barcode and primer sequences. Paired-end reads were merged using FLASH (version 1.2.7)^16^ (for details, see http://ccb.jhu.edu/software/FLASH). Quality filtering of the raw tags was performed under specific filtering conditions to obtain high-quality clean tags^17^ according to the QIIME (version 1.7.0)^18^ (for details, see http://qiime.org/scripts/split_libraries_fastq.html) quality control (QC) process.

The tags were compared with the reference database (Gold database, for details, see http://drive5.com/uchime/uchime_download.html) using the UCHIME algorithm (for details, see http://www.drive5.com/usearch/manual/uchime_algo.html)^19^ to detect chimera sequences (for details, see https://drive5.com/usearch/manual/chimeras.html); the chimera sequences were then removed^20^. Finally, the effective tags were obtained.

### OTU clustering and taxonomic annotation

Analysis of species diversity based on the sequencing data was performed by Uparse software (Uparse version 7.0.1001, for details, see http://drive5.com/uparse/)^21^ using all the effective tags. Sequences with ≥ 97% similarity were assigned to the same operational taxonomic units (OTUs). Representative sequences for each OTU were screened for further annotation. For each representative sequence, Mothur software was used with the SSUrRNA database of the SILVA Database (for details, see http://www.arb-silva.de/)^22^ for species annotation at each taxonomic rank (threshold: 0.8–1)^23^ (kingdom, phylum, class, order, family, genus, and species).

To study the microbial community composition, the bioinformatics data were first analyzed by applying a QIIME QC process to obtain clean data so that the results would be more accurate and reliable. The clean data were then processed by clustering based on 97% identity for the effective tags of all samples. According to the OTU clustering results, taxonomic annotation was performed for the representative sequence of each OTU to obtain the corresponding taxon information and taxon-based abundance distribution.

To obtain the phylogenetic relationship of all OTU representative sequences, MUSCLE^24^ (version 3.8.31, for details, see http://www.drive5.com/muscle/) was used to rapidly compare multiple sequences. OTU abundance information was normalized using a standard sequence number corresponding to that of the sample with the fewest sequences. Subsequent analyses of alpha diversity and beta diversity as higher-level measures were all performed based on these output-normalized data.

In addition, based on the OTU clustering results, we performed multiple sequence alignments and established phylogenetic trees. The differences between subjects’ fecal samples or among groups regarding the structure of the microbial community were explained via dimension reduction (principal coordinate analysis (PCoA), principal component analysis (PCA), and NMDS) and the unweighted pair group method with arithmetic mean (UPGMA). Statistical methods such as T-test, MetaStat, LEfSe, analysis of similarity (ANOSIM), and multiple response permutation procedure (MRPP) were used to test the significance of the microbial community structure differences among groups. Moreover, the results were subjected to canonical correlation analysis (CCA)/redundancy analysis (RDA) to explore the major environmental factors.

### Complexity of biodiversity and differences in species complexity

To analyze the complexity of the biodiversity of the microbiome, we determined the alpha diversity for a sample through calculation of several indices, including the observed species, Chao1, and Shannon indices. All these indices were calculated with QIIME (version 1.7.0) and displayed with R software (version 2.15.3)^25^.

To evaluate differences among samples in species complexity, we applied beta diversity analysis. Beta diversity based on both weighted and unweighted UniFrac distances was calculated by QIIME software (version 1.7.0).

Furthermore, cluster analysis preceded by PCA was applied to reduce the dimensions of the original variables using the FactoMineR (http://factominer.free.fr) package and ggplot2 package in R software (version 2.15.3)^25^. PCoA was performed to obtain principal coordinates and visualize complex, multidimensional data. A distance matrix of weighted or unweighted UniFrac distance among samples obtained in previous analysis was transformed to a new set of orthogonal axes, by which the maximum variation factor was demonstrated by the first principal coordinate, the second maximum variation factor was demonstrated by the second principal coordinate, and so on. PCoA results were displayed by the WGCNA, stat and ggplot2 packages in R software (version 2.15.3)^25^. UPGMA clustering was performed as a type of hierarchical clustering method to interpret the distance matrix using average linkage and was conducted by QIIME software (version 1.7.0).

MetaStat analysis was conducted by R software^25^. The *P*-value was calculated by the permutation test method, while the q-value was calculated by the Benjamini and Hochberg false discovery rate method^26^. ANOSIM, MRPP and Adonis were performed by R software (Vegan package: the ANOSIM function, MRPP function and adonis function, respectively)^25^. Analysis of molecular variance (AMOVA) was calculated by Mothur using the AMOVA function. T-tests and drawing were conducted by R software^25^.

### Quality control and limitations

Each step, such as sample testing, PCR, purification, library preparation and sequencing, affect the quality and quantity of data, while the data quality directly affects the subsequent information analysis results. To maintain the accuracy and reliability of sequencing data, QC was performed at each step of the procedure.

In our study, there were only limited samples (24 DNA samples obtained from 8 subjects) for NGS profiling; thus, statistical bias was possible.

### Ethical approval

All methods were carried out in accordance with relevant guidelines and regulations. We confirm that all experimental protocols were approved by the ethics committee of the Faculty of Medicine, Universitas Indonesia.

### Informed consent

We confirm that informed consent was obtained from all subjects’ legal guardian(s).

## Results

### Subjects, fecal samples, and clinical characteristics of neonates

All subjects were preterm inpatient neonates born in CMH, Jakarta. All fecal samples were collected from those eight subjects by performing the same procedure at three time points, i.e., meconium (24–48 h) and feces on day 4 and day 7, providing 24 samples. The subjects consisted of an equal number of male and female neonates who were late preterm and very preterm neonates, with gestational ages ranging from 30 to 34 weeks and birth weights ranging from 1080 to 1900 g. All subjects were born via C-section, except one born spontaneously; subject AJM. The frequencies of the feeding patterns breast milk only and mixed feeding were almost equal (5–3, respectively). There were more neonates who did not receive antibiotics than those who did, while during pregnancy, only two out of 8 subjects’ mothers received antibiotics. Total serum bilirubin (TSB) levels were all within the normal limit. These characteristics are shown in Table 1.

We also collected the dietary records of the mothers and found that the overall dietary pattern lacked dairy intake. The complete dietary pattern of the mothers is summarized in Supplementary Table 1.

### Microbial community composition

In the process of constructing OTUs for studying the microbial community composition, basic information about different samples was collected, such as effective tag data, low-frequency tag data and tag annotation data. A summary of this information is shown in Supplementary Fig. 1.

**Figure 1:**
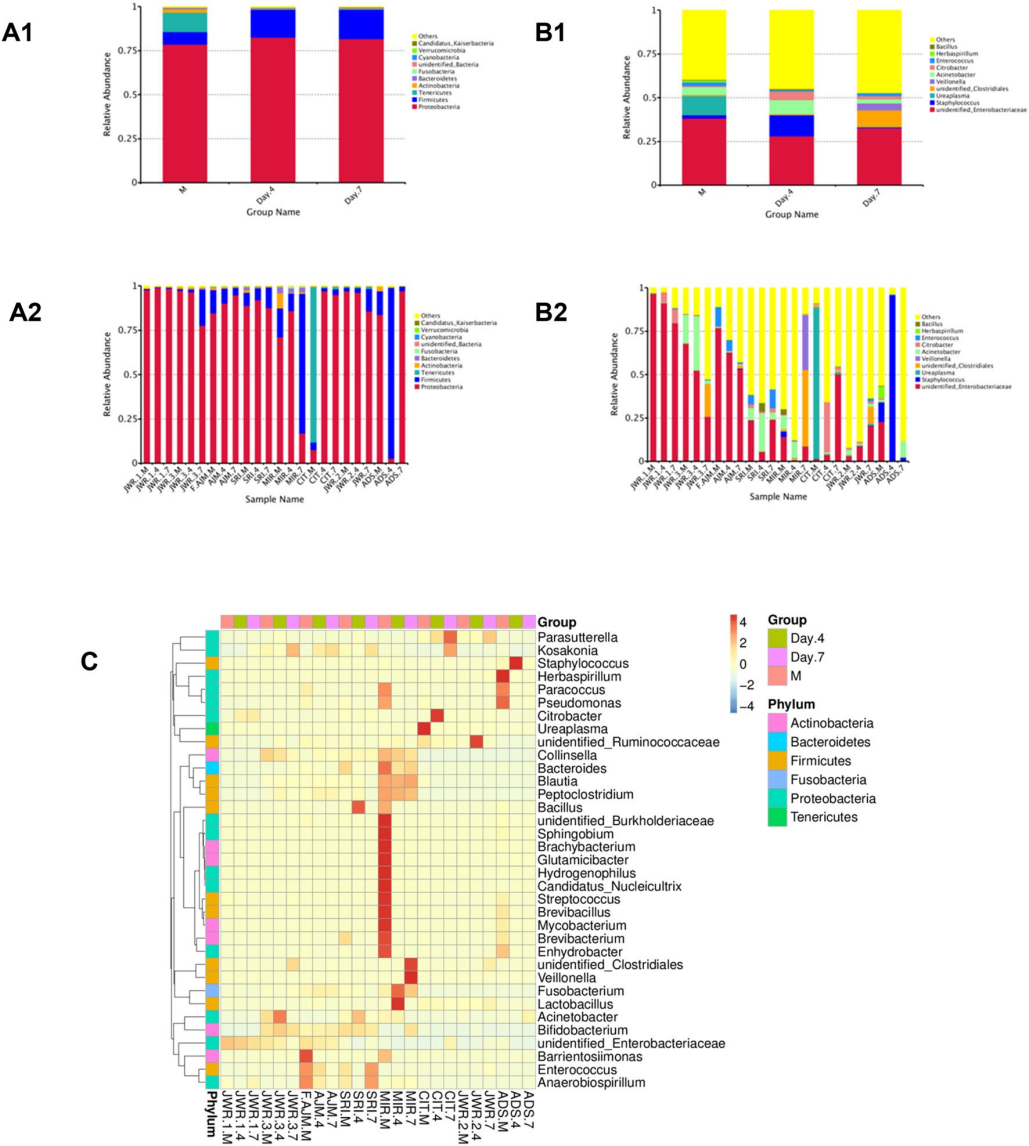
Analysis of OTUs and microbial composition in fecal samples at three time points for each subject. (**A1**) and (**A2**) show the results of analysis at the phylum level, whereas (**B1**) and (**B2**) show the results of analysis at the genus level, with all phylum and genus names designated in the figure legend inserts. (**A1**) and (**B1**) show the results for three time points, i.e., meconium (M), day 4 feces (Day 4) and day 7 feces (Day 7), while (**A2**) and (**B2**) show the three time points for each subject. All variation is shown in terms of relative abundance. (**C**) Heatmap of the relative abundance of 35 genera across three time points, with OTU proportions represented by bars. Generated by using R Software^24^.

The OTUs composing the microbial communities of fecal samples from the three time-point groups and each subject at the phylum and genus levels are summarized in Fig. 1. As observed in all groups and subjects, the order of abundance at the phylum level was Proteobacteria followed by Firmicutes (Fig. 1A1,A2), which agrees with previous reports^27–29^. At the genus level, unidentified *Enterobacteriaceae* was the most abundant (Fig. 1B1,B2). However, the second most abundant genus among these OTUs could not be determined, although *Staphylococcus* showed intriguing abundance in the fecal of one subject, ADS(4). In total, only 4 fecal samples from 2 subjects showed the presence of *Staphylococcus*, i.e., MIR(M), ADS(M), ADS(4), and ADS(7). Heatmap of 35 genera result gave great supportive data for OTUs analysis result at Genus level as presented in Fig. 1. It is interesting that relative abundance of *Ureaplasma* was found in CIT meconium, which disappeared in day 4 and day 7 fecal samples, which indicating the influence of maternal factor. Other interesting genus, *Lactobacillus*, was found in MIR day 4 fecal but was unable to develop as shown in day 7 feces.

Taxonomic annotation at the kingdom through the genus level of all subjects at the three time points is shown with column graphs in Supplementary Figs. 2–7. Taxonomic annotation at the kingdom through the species level for the time points was visualized with phylogenetic trees, as shown in Supplementary Figs. 8–10.

Taxonomic annotation for the 50 highest abundance OTUs in every time-point group is summarized in Table 2. The complete OTU taxonomic annotation at the species level of all time-point groups is summarized in Supplementary Table 2.

**Table 2:** 50 Highest OTU taxonomic annotations in species level of three time-point groups extracted from OTUs Taxonomy annotation list of species.

Meconium*		
*Escherichia_coli*	*Acinetobacter_ursingii*	*Achromobacter_xylosoxidans_subsp_xylosoxidans*
*Staphylococcus_haemolyticus*	*Serratia_marcescens*	*Mycobacterium_gordonae*
*Clostridium_perfringens*	*[Pseudomonas]_geniculata*	*Pseudomonas_peli*
*Acinetobacter_baumannii*	*Acinetobacter_schindleri*	*Lolium_perenne*
*Citrobacter_koseri*	*Collinsella_stercoris*	*Methylobacterium_aquaticum*
*Bacillus_anthracis*	*Sphingobacterium_multivorum*	*Romboutsia_sp_MT17*
*Streptococcus_gallolyticus_subsp_macedonicus*	*Acinetobacter_variabilis*	*Ralstonia_pickettii*
*Lactobacillus_reuteri*	*Proteus_mirabilis*	*Lachnospiraceae_bacterium_28-4*
*Clostridium_sp_ND2*	*Bacteroides_plebeius*	*Kocuria_rhizophila*
*Bacteroides_vulgatus*	*Micrococcus_luteus*	*Bifidobacterium_saeculare*
*Sphingobium_yanoikuyae*	*Collinsella_aerofaciens*	*Lachnospiraceae_bacterium_2_1_46FAA*
*Bacteroides_stercoris*	*Corynebacterium_tuberculostearicum*	*Chryseobacterium_hominis*
*Moraxella_osloensis*	*Clostridium_disporicum*	*Collinsella_tanakaei*
*Acinetobacter_junii*	*Helicobacter_bilis*	*Bacteroides_massiliensis*
*Blautia_sp_YL58*	*[Ruminococcus]_gnavus*	*butyrate-producing_bacterium_L2-10*
*Pseudomonas_stutzeri*	*Aeromonas_caviae*	
*Anaerobiospirillum_succiniciproducens*	*Gemella_haemolysans*	

Day 4 feces**		
*Escherichia_coli*	*[Clostridium]_papyrosolvens*	*Collinsella_tanakaei*
*Staphylococcus_haemolyticus*	*Acinetobacter_schindleri*	*Bacteroides_massiliensis*
*Clostridium_perfringens*	*Collinsella_stercoris*	*Campylobacter_helveticus*
*Acinetobacter_baumannii*	*Acinetobacter_variabilis*	*Parabacteroides_merdae*
*Citrobacter_koseri*	*Bacteroides_plebeius*	*butyrate-producing_bacterium_L2-10*
*Bacillus_anthracis*	*Planococcus_rifietoensis*	*Limnohabitans_curvus*
*Streptococcus_gallolyticus_subsp_macedonicus*	*Acinetobacter_lwoffii*	*Bacteroides_coprocola*
*Lactobacillus_reuteri*	*Collinsella_aerofaciens*	*Polynucleobacter_asymbioticus*
*Clostridium_sp_ND2*	*Corynebacterium_tuberculostearicum*	*Parasutterella_secunda*
*Bacteroides_vulgatus*	*Clostridium_disporicum*	*Bacteroides_thetaiotaomicron*
*Bacteroides_stercoris*	*Helicobacter_bilis*	*Blautia_hydrogenotrophica*
*Moraxella_osloensis*	*[Ruminococcus]_gnavus*	*Desulfovibrio_sp_ABHU2SB*
*Acinetobacter_junii*	*Romboutsia_sp_MT17*	*Rothia_mucilaginosa*
*Clostridium_butyricum*	*Planomicrobium_glaciei*	*Exiguobacterium_aurantiacum*
*Blautia_sp_YL58*	*Bifidobacterium_saeculare*	*Clostridiales_bacterium_CIEAF_020*
*Pseudomonas_stutzeri*	*Lachnospiraceae_bacterium_2_1_46FAA*	*Clostridium_argentinense*
*Anaerobiospirillum_succiniciproducens*	*Psychrobacter_alimentarius*	*[Eubacterium]_dolichum*

Day 7 feces***		
*Escherichia_coli*	*[Pseudomonas]_geniculata*	*Collinsella_tanakaei*
*Staphylococcus_haemolyticus*	*Acinetobacter_schindleri*	*Bacteroides_massiliensis*
*Clostridium_perfringens*	*Collinsella_stercoris*	*Campylobacter_helveticus*
*Acinetobacter_baumannii*	*Acinetobacter_variabilis*	*Clostridium_colicanis*
*Citrobacter_koseri*	*Bacteroides_plebeius*	*Parabacteroides_merdae*
*Bacillus_anthracis*	*Planococcus_rifietoensis*	*butyrate-producing_bacterium_L2-10*
*Streptococcus_gallolyticus_subsp_macedonicus*	*Acinetobacter_lwoffii*	*Pseudomonas_luteola*
*Lactobacillus_reuteri*	*Collinsella_aerofaciens*	*Mucispirillum_schaedleri*
*Clostridium_sp_ND2*	*Clostridium_disporicum*	*Burkholderiales_bacterium_YL45*
*Bacteroides_vulgatus*	*Helicobacter_bilis*	*Limnohabitans_curvus*
*Bacteroides_stercoris*	*[Ruminococcus]_gnavus*	*Bacteroides_coprocola*
*Acinetobacter_junii*	*Romboutsia_sp_MT17*	*Polynucleobacter_asymbioticus*
*Clostridium_butyricum*	*Planomicrobium_glaciei*	*Bacteroides_thetaiotaomicron*
*Blautia_sp_YL58*	*Lachnospiraceae_bacterium_28-4*	*Blautia_hydrogenotrophica*
*Pseudomonas_stutzeri*	*Bifidobacterium_saeculare*	*Rothia_mucilaginosa*
*Anaerobiospirillum_succiniciproducens*	*Lachnospiraceae_bacterium_2_1_46FAA*	*Parabacteroides_distasonis*
*Serratia_marcescens*	*Psychrobacter_alimentarius*	*Exiguobacterium_aurantiacum*

*50 Highest OTU taxonomic annotations in species level of meconium.

**50 Highest OTU taxonomic annotations in species level of day 4 feces.

****50 Highest OTU taxonomic annotations in species level of day 7 feces.

### Microbiome diversity and abundance in feces of the three time-point groups

**Figure 2:**
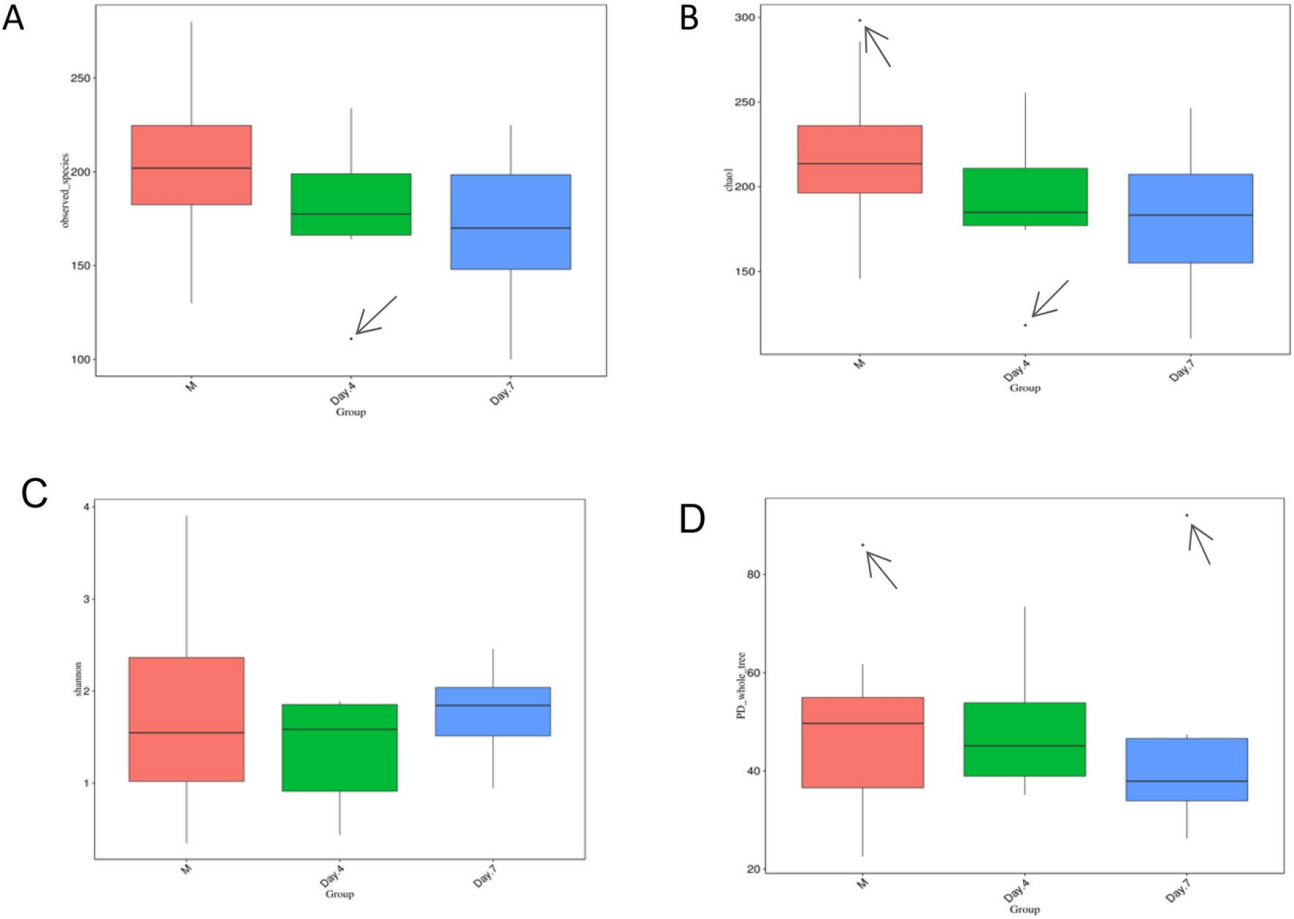
Differences in alpha diversity indices among groups shown by a boxplot. (**A**) Observed species, (**B**) Shannon index, (**C**) Chao1 index, and (**D**) Faith’s phylogenetic diversity across three time points. Boxes represent the interquartile ranges (IQRs) among M, Day 4, and Day 7, while the horizontal lines inside the boxes define the medians. Whiskers represent the lowest and highest values within 1.5 times the IQR. Small circle symbols in (**A**), (**C**), and (**D**) indicate outliers (values greater than 1.5 times and less than three times the IQR). Generated by using R Software^24^.

The richness and evenness information about the microbiome diversity across three time-point groups, and common and unique OTU information among different groups were obtained by using alpha diversity assessment. This measure allowed us to assess a broader change or difference in the composition of microorganisms for describing the microbiome. Based on alpha diversity assessments data performed, i.e., observed species, Shannon diversity, and chao1 diversity, as well as Faith’s Phylogenetic Diversity across three-time points groups (meconium, day 4 and day 7) as presented in Fig. 2, significantly different composition of microorganisms for describing the microbiome between groups was not observed. It demonstrated that the pattern of early life microbiome development exhibited similar species richness and evenness in three-time points groups, although with some exceptions for unhealthy neonates represented by outliers’ value.

A rank abundance curve was made to sort the OTUs in the sample by relative abundance (or the number of sequences included) from greatest to least to obtain the corresponding sorting number, which can be used as the ordinate. The observed OTUs, Chao1 index, Shannon index, and Faith’s phylogenetic diversity (PD) were generated from the alpha diversity assessment with boxplot visualization, as shown in Fig. 2. There were outliers in the alpha diversity indices observed species (Fig. 2A) on day 4, Chao1 diversity (Fig. 2C) in M and on day 4, and Faith’s PD (Fig. 2D) in M and on day 7.

We produced alpha rarefaction curves to indicate whether the biodiversity of microbiome samples reflects the representativeness of the sequencing data directly and reflects the richness of the microbial community in the samples indirectly. The curve flattened (Supplementary Fig. 11), indicating that a reliable number of samples was used, which means that only scarce species remained to be sampled.

### Microbiome composition changes in feces at the three time points

**Figure 3:**
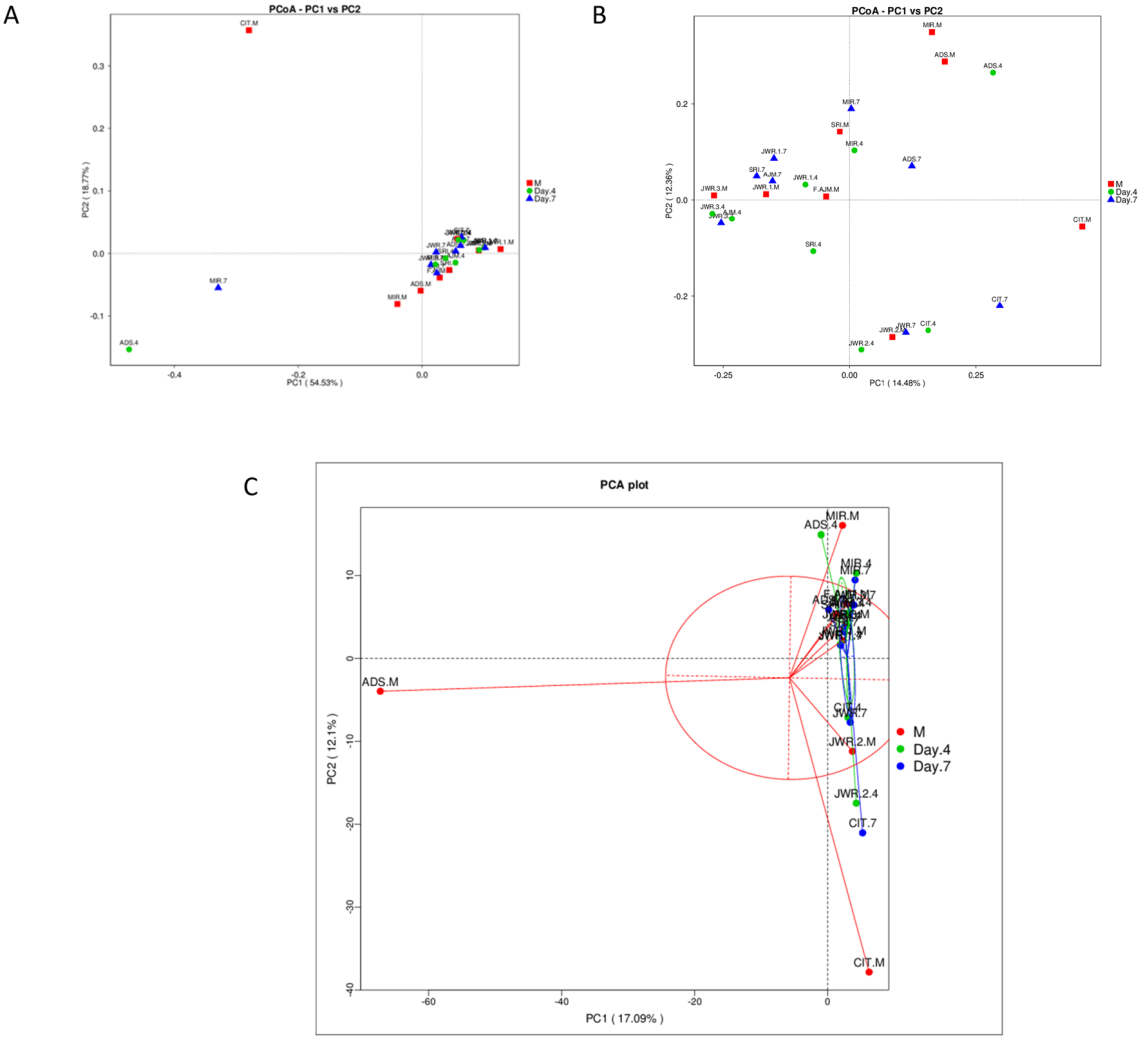
Beta diversity analysis results displayed by a PCoA plot based on (**A**) weighted UniFrac (**B**) unweighted UniFrac distances. (**C**) Shows a PCA plot. Generated by using R Software^24^.

Beta diversity measures were assessed to capture changes in microbiome community composition among the groups. The dissimilarity among groups was calculated with gradient analysis and displayed with ordination plots (PCA, PCoA, etc.). Samples with similar microbial community structures tended to cluster, and vice versa. Here, we used the PCoA method with weighted UniFrac and unweighted UniFrac distances and visualized the results as a graph, as shown in Fig. 3.

Furthermore, samples were clustered by UPGMA based on the acquired distance matrix to construct a cluster tree (Supplementary Figs. 12, 13). Thus, with the UPGMA tree, which considered high-abundance taxa to achieve the underlying driving factors, we were able to determine the complexity differences between day 4 and day 7, which were fewer than those between M and either postnatal group.

To learn more about the development of the microbiome community over the first week in newborn preterm neonates’ feces based on their changes and differences, we used ANOSIM. As a nonparametric test, ANOSIM could evaluate whether the variation in the feces microbiome among time-point groups was significantly larger than the variation within groups, i.e., early, day 4, and day 7; this information helped us evaluate the reasonability of the division of groups.

**Figure 4:**
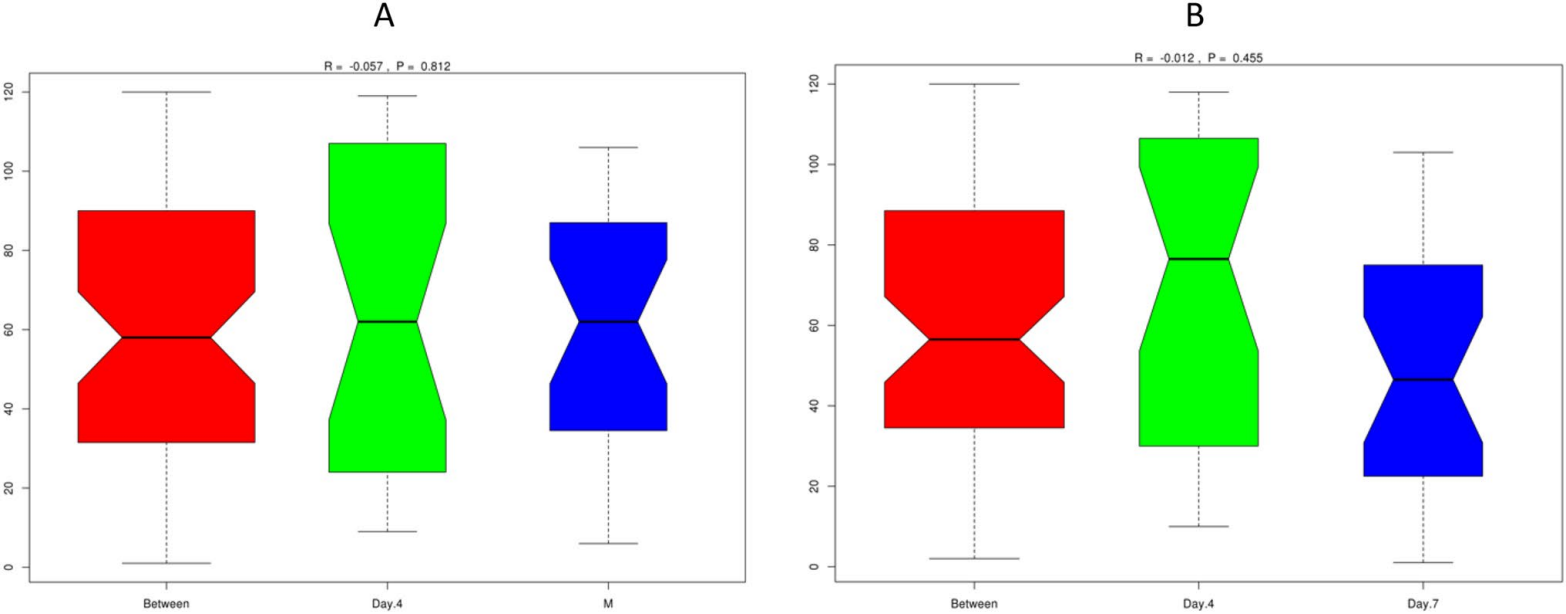
ANOSIM results for the feces microbiome at the three time points. Unweighted UniFrac distances between microbiome communities of subjects’ feces within the three time points, for comparisons between each of the two groups, i.e., M with day 4 (**A**) and day 4 with day 7 (**B**). No significant differences were observed (high *P*-values, 0.812 and 0.455). Generated by using R Software^24^.

Our results showed a negative R-value, which suggested that intragroup variation was larger than intergroup variation. Therefore, there were no significant differences between early (M) and day 4 (Fig. 4A) or day 4 and day 7 (Fig. 4B), with *P* = 0.812 and *P* = 0.455, respectively. Boxplots based on the rank (between-group and within-group) of the ANOSIM results are shown in Fig. 4. The rank was obtained from the sorted distance between groups.

**Figure 5:**
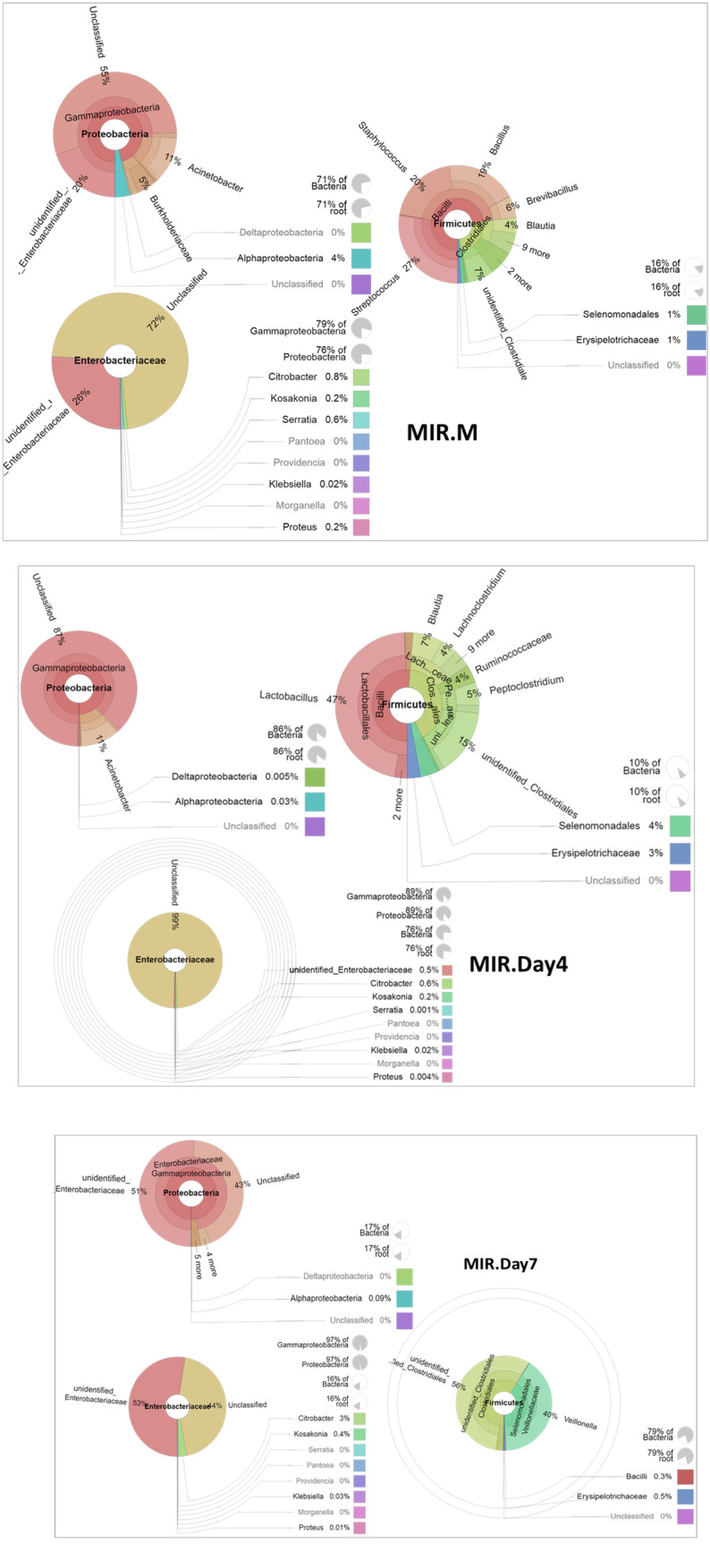
The changes in and development of the microbiome diversity and proportions of subject MIR feces at the three time points, i.e., the MIR.M, MIR.Day4 and MIR.Day7 samples, shown by Krona displays, revealing the most abundant phylum Proteobacteria, followed by the most abundant genus *Enterobacteriaceae* and the second most abundant phylum Firmicutes. In the phylum Firmicutes in the MIR.Day4 and MIR.Day7 fecal samples, interesting genera were found, i.e., *Lactobacillus* and *Veillonella* spp., respectively. Generated by using R Software^24^.

A Krona plot displayed the microbiome diversity and proportion in the feces of subject MIR at the three time points, is shown in Fig. 5.

The microbiome diversity and proportion of these triplets are shown as circular representations by Krona displays in Supplementary Fig. 14.

**Figure 6:**
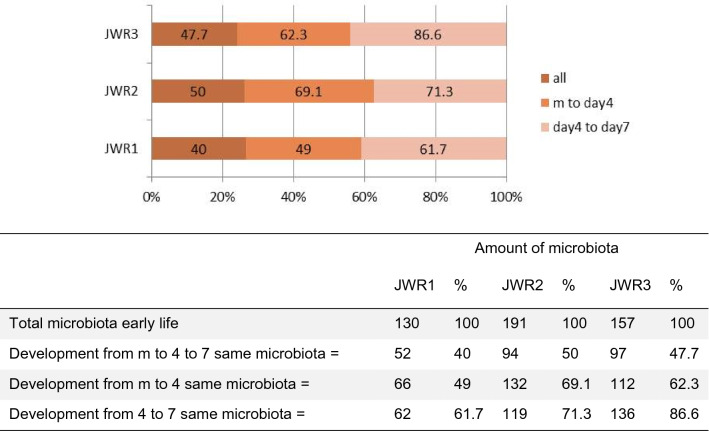
The development of the feces microbiome of the triplet subjects from early life to one week later, showing the microbiome shared by M and Day 4 and by Day 4–7 samples, as presented in a Venn diagram (Supplementary Fig. 14). The proportion (percentage) of the same microbiome diversity is summarized as a horizontal bar, and the matrix is underneath (below).

The development of the feces microbiome of the triplet subjects from meconium, day 4, and day 7 with their proportion is shown in Fig. 6.

## Discussion

This study shows the development and diversity of the gut microbiome in 24 samples derived from the feces samples of eight preterm neonates born at CMH, Jakarta, at 3 different time points in early life. The analysis of gut microbiome development over the first week based on alpha and beta diversity in terms of relative abundance showed that for the 8 subjects providing 24 feces samples at the three time points, i.e., meconium, day 4 feces, and day 7 feces, Proteobacteria and Firmicutes were dominant at the phylum level, while at the genus level, unidentified *Enterobacteriaceae* were most abundant, as has been reported widely^1,6,7,9,10,29–34^. This result is similar to our previous study on cultivable bacteria from meconium from neonates born in CMH^9^. The microbiomes of meconium, as the first feces in this study, were relatively similar, with one subject showing a high abundance of the genus *Ureaplasma* and the rest showing a high abundance of Enterobacter.

The meconium microbiome shows different progression and development in preterm neonates, as reported, even among triplets, as revealed in this study^5,11,27,30,31,35,36^. The meconium microbiome from our study was not significantly affected by perinatal factors. The neonatal gut microbiome is known to begin colonization in utero and is affected by genetic and maternal factors during pregnancy, including chorioamnionitis^13,37,38^.

Raw data were processed to obtain clean data prior to OTU analysis of paired-end sequencing reads and then merged by using FLASH, a very fast and accurate analysis tool, which was designed to merge paired-end reads when at least some of the reads overlapped the reads generated from the opposite end of the same DNA fragment, and the splicing sequences were called raw tags. Based on the OTU analysis of the microbial composition (Fig. 1), one patient (CIT) showed *Ureaplasma* as the most abundant taxon in the meconium (Fig. 1B2), which can be explained by the fact that the mother had premature rupture of membranes (PROM) over 18 h, which is a risk factor for chorioamnionitis (Table 1). Moreover, OTU analysis of the ADS and SRI samples showed proportional diversity among each abundant genus (Fig. 1B2). These two patients’ mothers had leukocytosis as a risk factor for chorioamnionitis but without PROM. This observation could be explained by how the microbiome can be directly affected by chorioamnionitis, specifically by the incidence of PROM, not by other risk factors such as only leukocytosis. It is shown that PROM could directly expose the fetal digestive system to foreign bacteria during systemic infection, whereas the risk of direct contamination is far less^1–5,29,30,39^. This emphasizes the importance of maintaining infection prevention during pregnancy but, more importantly, prompt delivery when indicated for PROM cases.

Diversity of the meconium microbiome reflects the overall quality of the fetus and the mother^4,5,29,38^. In this case, MIR was born the most maturely (at 34 weeks, see Table 1) among the subjects, with an optimum Apgar score and no risk of chorioamnionitis. This background is in accordance with having a diverse meconium microbiome, showing the importance of maintaining both a healthy mother and fetus, which will affect the immediate future of the neonate upon birth, especially regarding feeding tolerance and response to infection^4,9,30,35^.

Subject MIR was the neonate with the best conditions not only prenatally but also postnatally. *Veillonella* spp. were detected in this subject’s microbiome at day 7. *Veillonella* spp. are part of several normal human microbiome, including the fecal microbiome of healthy adults, and are predominant in the upper digestive tract^40,41^. Variations in the counts of *Veillonella* spp. have previously been noted depending on diet, pathology, or antimicrobial treatment^42,43^. *Veillonella* may play a role in gut microbiome development at an early age since it has been shown to appear in neonate feces in the first days of life, with a significantly higher count in bottle-fed than in breast-fed children^42–44^.

The pattern of development began with Enterobacter, which was detected initially in meconium, and then changes depending on the environmental factors. Antibiotics were administered due to the high risk of early-onset neonatal sepsis that occurs in preterm neonates affected by perinatal factors^45,46^. The regimen given is usually based on the microbe map of the particular center. In our study, 6 out of 8 patients were given first-line antibiotics (ampicillin and gentamicin). Only three of these patients developed a non-Enterobacter-dominant microbiome on day 4 or day 7, which shows that we were unable to conclude whether early life administration of first-line antibiotics have a significant effect on the early life gut microbiome from our study. One interesting case was with triplet patients, i.e., JWR1 (first triplet), JWR2 (second triplet) and JWR3 (third triplet) which had varying populations of microbiome. Two of the triplets had non-Enterobacter-dominant microbiomes. From a blood culture of the first triplet (JWR1), *Escherichia coli* was detected; however, the microbiome was dominated by the phylum Citrobacter. The third triplet (JWR3) had Acinetobacter and Clostridium as the dominant microbiome constituents at day 4 and day 7, respectively, with no blood culture growth. All three of the triplets had feeding intolerance, with the worst occurring in the third triplet (JWR3) (Supplementary Fig. 14). This result differs with a previous study comparing dichorionic triplets which showed a significantly strong similarity of microbiome between triplets^47^. This disparity can be due to the smaller triplet in which reflects a worse state of prematurity including the intestinal permeability, therefore directly affecting the early life gut microbiome.

The direct effect of bacteremia on the gut microbiome is significant, especially in cases of late-onset neonatal sepsis; in addition, neonatal sepsis will detrimentally impact the already premature immune system and contribute indirectly to the abundance of non-commensal bacteria in the gut, such as *Acinetobacter*, *Citrobacter*, and *Clostridium*^48,49^. The administration of antibiotics, especially those that target anaerobes, can also eliminate commensal normal flora bacteria and overgrowth of pathogenic bacteria such as *Clostridium difficile*, thus causing antibiotic-associated diarrhea^50^. This effect usually occurs after 14 days of antibiotic administration. However, in cases of under intensive care and immunosuppression, as well as a premature gut microbiome, gut dysbiosis can occur much earlier^28,30,33,51,52^. Our study showed that the triplets who received the same antibiotics, 2 of the triplets (JWR2 and JWR3) showed varying microbiome populations, most likely due to worsening bacteremia.

Another interesting finding is the diversity among the feces microbiome from the three time points. The diversity lessens between each time point; however, the samples shared the same species at certain amounts, in addition, complexity of day 4 and day 7 is less than that of meconium compared to day 4 and day 7. As visualized by a Venn diagram (Supplementary Fig. 15), the meconium microbiome exhibited the same species richness and evenness in those subjects, which may be considered consistent intestinal homeostasis in preterm neonates. For example, microbiome development in the case of the triplets (JWR1, JWR2 and JWR3), from early life to one week later, showed that the microbiome shared common microbiome population early life (meconium) and at day 4, while that at day 4 shared common microbiome population with that at day 7 and influenced by nutrition intake, as summarized in Fig. 6.

This observation can be explained by the mechanism of microbial maturation and colonization^2,11,13,53^. Prenatally, the gut is heavily and dominantly influenced by the genetic determinants, only after the postnatal period, environmental factors including those that affect the brain development confers the dominant influence^2,29,30,33^. The postnatal developmental phases of the gut and brain are interdependent and occur separately. The developmental interdependency between the gut and brain is due to the complex, bidirectional communication, incorporating neural, endocrine, immune, and metabolic mediators termed GBA^54^. Several ways the GBA works if via both afferent and efferent autonomic pathways with different intestinal targets such as enteric nervous system (ENS), muscle layers and gut mucosa, modulating motility, immunity, permeability, and secretion of mucus; which results in the development and alteration of the gut microbiome^2,29,30,33,54^. During the process on the 4^th^ day, exposure of the neonate to the environment, intake of medicine and enteral/parenteral nutrition, and influences of insults that affect the innate immune system shifted the microbiome into a less diverse population. By the 7th day, the microbiome further shifted into specific populations, albeit the shift was not as significant as the shift from meconium to feces on the 4th day. There were some differences in colonization, possibly due to the comorbidities of the neonates, such as bacteremia and antibiotic administration. However, the detrimental dysbiosis effect due to antibiotic use usually occurs after administration for longer than 14 days; therefore, in our case, a more likely explanation is based on bacteremia, neonate prematurity, and lack of enteral supply of probiotics (e.g., mother’s milk)^3,13,33,38,55–57^. Despite observing differences among the time points, the differences were not statistically significant. Meconium and day 7 feces should display tremendous differences, but the difference had a *P*-value of only 0.612. This may be due to the small number of samples not being sufficient to perform an optimal statistical analysis.

Surprisingly, we did not find any *Bifidobacterium* or *Lactobacillus* abundant in our study. Maternal, perinatal, and postnatal factors should influence the normal microbiome. From the food survey of the mothers, we found that all the mothers’ dairy consumption was lower than recommended (Supplementary Table 1). *Bifidobacterium* and *Lactobacillus* are acquired mainly from intake of dairy products. The abundance among the maternal population of those microbiome constituents greatly influences the fetal microbiome^58^. Even by the 7th day, the microbiome population of these neonates did not reach adequate abundance of *Bifidobacterium* and *Lactobacillus*, mainly due to both the lack of dairy consumption and postnatal exposure to probiotics, which are usually acquired from mother’s milk. This phenomenon should be further studied to determine whether we can optimize the neonate’s innate immune response and early tolerance to feeding by inducing earlier colonization of beneficial gut microbiome constituents using enhanced preterm formula milk or by any other means.

The limitation of our study, first and foremost, is the sample collection method. We were unable to collect fresh meconium samples to prevent contamination. The reason for this is that the method to obtain fresh meconium in preterm neonates requires administration of an anal spool using saline or glycerol. This technique is potentially harmful due to the nature of preterm gastrointestinal function in which peristaltic movement is usually delayed; therefore, collecting the sample directly and not from the diapers is of utmost difficulty if not actively monitored. The results were still within acceptable limits, whereas contamination would yield lower abundance from the DNA sequencing and would still yield a positive result, but the most dominant genus would still not be from the contamination source. Our second limitation is the small number of samples. We originally opted to collect more samples; however, this work serves as a preliminary study on how the microbiome of preterm neonates in our center is established. We deem the results of the metagenomic analysis to be acceptable for use as the basis for future studies.

## Conclusion

A myriad of factors influences the development of the neonatal gut microbiome, including maternal, perinatal, and postnatal factors. Indonesian pregnant women have markedly low consumption of dairy products, which results in decreased populations of *Bifidobacterium* and *Lactobacillus*. The most abundant genus was unidentified *Enterobacteriaceae*, with slight variations in specific samples dominated by *Clostridiales* and *Ureaplasma,* and *Proteobacteria* and *Firmicutes* showed dominance at the phylum level. Perinatal factors showed influence on the meconium microbiome including PROM and mother’s diet, in which populations of *Bifidobacterium* and *Lactobacillus* was not found, instead *Ureaplasma* was one of the dominant populations in the patient with mother’s history of PROM. Notable associations between early life fecal microbiome and clinical outcomes include administration of early antibiotics and bacteremia, with the ensuing microbiome population to show less diversity. The development of the preterm neonate gut microbiome from meconium to day 4 and day 7 showed a pattern of change in diversity as time progresses, but a similarity with the meconium microbiome was maintained, and with decreasing complexity. These data emphasize the need for specific evaluation and management of dysbiosis and their related clinical outcomes in different populations.

## Supplementary Information

Supplementary Information.

## Data Availability

The datasets used and/or analysed during the current study is available from the corresponding author on reasonable request.

## Abbreviations

CMH: Cipto Mangunkusumo Hospital
DNA: Deoxyribonucleic acid
GBA: Gut-brain axis
IQR: Interquartile range
NICU: Neonatal intensive care unit
NGS: Next-generation sequencing
OTU: Operational taxonomic unit
PCA: Principal component analysis
PCoA: Principal coordinate analysis
PCR: Polymerase chain reaction
QC: Quality control
UPGMA: Unweighted pair group method with arithmetic mean
WGCNA: Weighted correlation network analysis
