# Supplementary material for: Metagenomic profiles of the early life microbiome of Indonesian inpatient neonates and their influence on clinical characteristics
Source: Sci Rep. 2022 Jun 7;12:9413. doi: 10.1038/s41598-022-13496-4 (PMC9174262; doi:10.1038/s41598-022-13496-4)

Supplementary Table 1. Diet pattern of the subjects’ mothers

|  | 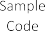 | 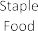 |  | 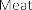 | 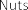 | 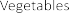 | 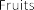 |  | 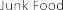 | 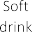 | 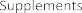 | 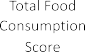 |
| --- | --- | --- | --- | --- | --- | --- | --- | --- | --- | --- | --- | --- |
|  |  | 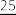 |  |  |  |  |  |  |  |  |  |  |
|  |  |  |  |  |  |  |  |  |  |  |  |  |
|  |  |  |  |  |  |  |  |  |  |  |  |  |
|  |  |  |  |  |  |  |  |  |  |  |  |  |
|  |  |  |  |  |  |  |  |  |  | 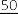 |  |  |

Supplementary Table 2. Complete OTUs taxonomic annotation in species level of all time-point groups

| Taxonomy | M | Day.4 | Day.7 |
| --- | --- | --- | --- |
| 1 Escherichia_coli | 0.382373 | 0.280217 | 0.327654 |
| 2 Staphylococcus_haemolyticus | 0.018526 | 0.120378 | 0.003990 |
| 3 Clostridium_perfringens | 0.005034 | 0.004011 | 0.090722 |
| 4 Acinetobacter_baumannii | 0.042664 | 0.080284 | 0.022100 |
| 5 Citrobacter_koseri | 0.006487 | 0.047215 | 0.018512 |
| 6 Bacillus_anthracis | 0.004252 | 0.005994 | 0.000124 |
| 7 Streptococcus_gallolyticus_subsp_macedonicus | 0.006653 | 0.000069 | 0.000034 |
| 8 Lactobacillus_reuteri | 0.000183 | 0.005524 | 0.000189 |
| 9 Clostridium_sp_ND2 | 0.000319 | 0.000240 | 0.002866 |
| 10 Bacteroides_vulgatus | 0.002222 | 0.000475 | 0.000570 |
| 11 Sphingobium_yanoikuyae | 0.001045 | 0.000004 | 0.000003 |
| 12 Bacteroides_stercoris | 0.001008 | 0.000236 | 0.000256 |
| 13 Moraxella_osloensis | 0.001250 | 0.000032 | 0.000005 |
| 14 Acinetobacter_junii | 0.000999 | 0.000045 | 0.000166 |
| 15 Clostridium_butyricum | 0.000023 | 0.000027 | 0.000674 |
| 16 Blautia_sp_YL58 | 0.000539 | 0.000573 | 0.000791 |
| 17 Pseudomonas_stutzeri | 0.000946 | 0.000017 | 0.000018 |
| 18 Anaerobiospirillum_succiniciproducens | 0.000896 | 0.000364 | 0.000794 |
| 19 Acinetobacter_ursingii | 0.000540 | 0.000019 | 0.000001 |
| 20 [Clostridium]_papyrosolvens | 0.000039 | 0.000484 | 0.000007 |
| 21 Serratia_marcescens | 0.000918 | 0.000009 | 0.000029 |
| 22 [Pseudomonas]_geniculata | 0.000523 | 0.000002 | 0.000016 |
| 23 Acinetobacter_schindleri | 0.000554 | 0.000125 | 0.000174 |

| 24 Collinsella_stercoris | 0.000704 | 0.000733 | 0.000490 |
| --- | --- | --- | --- |
| 25 Sphingobacterium_multivorum | 0.000319 | 0.000001 | 0.000000 |
| 26 Acinetobacter_variabilis | 0.000518 | 0.000032 | 0.000130 |
| 27 Proteus_mirabilis | 0.000386 | 0.000014 | 0.000004 |
| 28 Bacteroides_plebeius | 0.000298 | 0.000211 | 0.000339 |
| 29 Micrococcus_luteus | 0.000436 | 0.000010 | 0.000004 |
| 30 Planococcus_rifietoensis | 0.000000 | 0.000193 | 0.000226 |
| 31 Acinetobacter_lwoffii | 0.000000 | 0.000216 | 0.000158 |
| 32 Collinsella_aerofaciens | 0.000418 | 0.000339 | 0.000232 |
| 33 Corynebacterium_tuberculostearicum | 0.000597 | 0.000066 | 0.000011 |
| 34 Clostridium_disporicum | 0.000136 | 0.000250 | 0.000160 |
| 35 Helicobacter_bilis | 0.000372 | 0.000133 | 0.000258 |
| 36 [Ruminococcus]_gnavus | 0.000399 | 0.000264 | 0.000149 |
| 37 Aeromonas_caviae | 0.000188 | 0.000009 | 0.000013 |
| 38 Gemella_haemolysans | 0.000177 | 0.000013 | 0.000004 |
| 39 Achromobacter_xylosoxidans_subsp_xylosoxidans | 0.000172 | 0.000000 | 0.000002 |
| 40 Mycobacterium_gordonae | 0.000152 | 0.000000 | 0.000000 |
| 41 Pseudomonas_peli | 0.000153 | 0.000002 | 0.000013 |
| 42 Lolium_perenne | 0.000171 | 0.000005 | 0.000002 |
| 43 Methylobacterium_aquaticum | 0.000179 | 0.000006 | 0.000010 |
| 44 Romboutsia_sp_MT17 | 0.000131 | 0.000236 | 0.000183 |
| 45 Planomicrobium_glaciei | 0.000000 | 0.000102 | 0.000117 |
| 46 Ralstonia_pickettii | 0.000115 | 0.000002 | 0.000010 |
| 47 Lachnospiraceae_bacterium_28-4 | 0.000117 | 0.000004 | 0.000033 |
| 48 Kocuria_rhizophila | 0.000129 | 0.000002 | 0.000005 |
| 49 Bifidobacterium_saeculare | 0.000146 | 0.000078 | 0.000079 |
| 50 Lachnospiraceae_bacterium_2_1_46FAA | 0.000112 | 0.000241 | 0.000071 |
| Staphylococcus_lentus | 0.000099 | 0.000002 | 0.000000 |
| Chryseobacterium_hominis | 0.000160 | 0.000000 | 0.000000 |
| Psychrobacter_alimentarius | 0.000000 | 0.000090 | 0.000091 |
| Prevotella_pallens | 0.000093 | 0.000005 | 0.000005 |
| Lactobacillus_intestinalis | 0.000087 | 0.000005 | 0.000011 |
| Collinsella_tanakaei | 0.000192 | 0.000146 | 0.000174 |
| Cellulosimicrobium_cellulans | 0.000080 | 0.000001 | 0.000000 |
| Bacteroides_massiliensis | 0.000039 | 0.000108 | 0.000104 |
| Campylobacter_helveticus | 0.000115 | 0.000086 | 0.000102 |
| Clostridium_colicanis | 0.000020 | 0.000007 | 0.000101 |
| Corynebacterium_variabile | 0.000097 | 0.000003 | 0.000007 |
| Parabacteroides_merdae | 0.000084 | 0.000044 | 0.000030 |
| butyrate-producing_bacterium_L2-10 | 0.000117 | 0.000056 | 0.000022 |
| Pseudomonas_luteola | 0.000078 | 0.000014 | 0.000015 |
| Mucispirillum_schaedleri | 0.000086 | 0.000011 | 0.000032 |
| Burkholderiales_bacterium_YL45 | 0.000007 | 0.000016 | 0.000059 |

| Corynebacterium_appendicis | 0.000062 | 0.000000 | 0.000001 |
| --- | --- | --- | --- |
| Lachnospiraceae_bacterium_3-1 | 0.000056 | 0.000012 | 0.000013 |
| [Clostridium]_leptum | 0.000076 | 0.000009 | 0.000013 |
| Limnohabitans_curvus | 0.000000 | 0.000045 | 0.000017 |
| Acidovorax_delafieldii | 0.000044 | 0.000000 | 0.000003 |
| Bacteroides_coprocola | 0.000050 | 0.000043 | 0.000052 |
| Deinococcus_geothermalis | 0.000058 | 0.000000 | 0.000000 |
| Polynucleobacter_asymbioticus | 0.000000 | 0.000043 | 0.000030 |
| Parasutterella_secunda | 0.000004 | 0.000043 | 0.000014 |
| Bacteroides_thetaiotaomicron | 0.000085 | 0.000055 | 0.000075 |
| Blautia_hydrogenotrophica | 0.000015 | 0.000038 | 0.000052 |
| Ureibacillus_thermosphaericus | 0.000031 | 0.000000 | 0.000000 |
| Desulfovibrio_sp_ABHU2SB | 0.000002 | 0.000036 | 0.000007 |
| Pseudomonas_thermotolerans | 0.000030 | 0.000000 | 0.000000 |
| Rothia_mucilaginosa | 0.000025 | 0.000021 | 0.000050 |
| Corynebacterium_lipophiloflavum | 0.000030 | 0.000000 | 0.000000 |
| Parabacteroides_distasonis | 0.000042 | 0.000016 | 0.000038 |
| Burkholderia_sp_symbiont_of_Dicranocephalus_albip  es | 0.000023 | 0.000000 | 0.000000 |
| Lachnospiraceae_bacterium_615 | 0.000040 | 0.000012 | 0.000020 |
| Exiguobacterium_aurantiacum | 0.000003 | 0.000020 | 0.000025 |
| Idiomarina_sp | 0.000000 | 0.000000 | 0.000020 |
| Tyzzerella_sp_Marseille-P3062 | 0.000023 | 0.000007 | 0.000022 |
| Clostridiales_bacterium_CIEAF_020 | 0.000027 | 0.000017 | 0.000013 |
| Brevundimonas_vesicularis | 0.000019 | 0.000000 | 0.000000 |
| Xanthobacter_flavus | 0.000019 | 0.000000 | 0.000003 |
| Helicobacter_ganmani | 0.000018 | 0.000012 | 0.000009 |
| Lachnospiraceae_bacterium_A2 | 0.000001 | 0.000004 | 0.000018 |
| Clostridiales_bacterium_canine_oral_taxon_123 | 0.000005 | 0.000010 | 0.000020 |
| Epipyxis_sp_PR26KG | 0.000000 | 0.000000 | 0.000017 |
| Bradyrhizobium_elkanii | 0.000020 | 0.000009 | 0.000010 |
| Shewanella_putrefaciens | 0.000000 | 0.000013 | 0.000018 |
| Clostridium_argentinense | 0.000015 | 0.000034 | 0.000013 |
| Bifidobacterium_pseudocatenulatum | 0.000000 | 0.000007 | 0.000019 |
| Thermobispora_bispora | 0.000015 | 0.000000 | 0.000000 |
| Pseudoxanthomonas_taiwanensis | 0.000014 | 0.000000 | 0.000000 |
| [Eubacterium]_dolichum | 0.000029 | 0.000026 | 0.000012 |
| Sporosarcina_thermotolerans | 0.000000 | 0.000013 | 0.000000 |
| Lachnospira_pectinoschiza | 0.000013 | 0.000001 | 0.000003 |
| Candidatus_Nitrosoglobus_terrae | 0.000000 | 0.000013 | 0.000000 |
| Turicibacter_sanguinis | 0.000013 | 0.000011 | 0.000005 |
| Lachnospiraceae_bacterium_DW52 | 0.000000 | 0.000013 | 0.000000 |
| Nitrosomonas_sp | 0.000000 | 0.000011 | 0.000000 |

| Pseudomonas_anguilliseptica | 0.000000 | 0.000011 | 0.000007 |
| --- | --- | --- | --- |
| Brevundimonas_subvibrioides | 0.000000 | 0.000011 | 0.000007 |
| Lachnospiraceae_bacterium_COE1 | 0.000013 | 0.000003 | 0.000001 |
| Aquabacterium_citratiphilum | 0.000011 | 0.000000 | 0.000000 |
| bacterium_QTYC46b | 0.000000 | 0.000010 | 0.000000 |
| Butyricicoccus_pullicaecorum | 0.000002 | 0.000016 | 0.000015 |
| Prevotella_nanceiensis | 0.000000 | 0.000010 | 0.000000 |
| Saccharomonospora_viridis | 0.000010 | 0.000000 | 0.000000 |
| Clostridiales_bacterium_canine_oral_taxon_260 | 0.000000 | 0.000009 | 0.000000 |
| Clostridium_sp_ASF356 | 0.000013 | 0.000011 | 0.000005 |
| Pseudomonas_formosensis | 0.000009 | 0.000000 | 0.000000 |
| Veillonella_sp_oral_taxon_780 | 0.000000 | 0.000009 | 0.000000 |
| Cellvibrio_japonicus | 0.000007 | 0.000000 | 0.000000 |
| Clostridium_sp_ASF502 | 0.000002 | 0.000012 | 0.000004 |
| Bacillus_coagulans | 0.000007 | 0.000000 | 0.000001 |
| Leptotrichia_hofstadii | 0.000000 | 0.000006 | 0.000000 |
| Pseudomonas_azotoformans | 0.000007 | 0.000016 | 0.000013 |
| Exiguobacterium_undae | 0.000001 | 0.000000 | 0.000005 |
| Peptoniphilus_methioninivorax | 0.000000 | 0.000005 | 0.000002 |
| [Clostridium]_spiroforme | 0.000000 | 0.000000 | 0.000005 |
| Corynebacterium_amycolatum | 0.000005 | 0.000000 | 0.000000 |
| Blautia_coccoides | 0.000006 | 0.000000 | 0.000005 |
| Tetraselmis_cordiformis | 0.000000 | 0.000002 | 0.000005 |
| Paenibacillus_barengoltzii | 0.000005 | 0.000000 | 0.000000 |
| Haemophilus_parainfluenzae | 0.000003 | 0.000006 | 0.000000 |
| Rhodococcus_fascians | 0.000000 | 0.000004 | 0.000000 |
| [Clostridium]_colinum | 0.000007 | 0.000002 | 0.000000 |
| Lactococcus_lactis | 0.000004 | 0.000000 | 0.000002 |
| Nocardioides_marinus | 0.000004 | 0.000000 | 0.000000 |
| Comamonas_testosteroni | 0.000010 | 0.000000 | 0.000004 |
| Lactobacillus_iners | 0.000000 | 0.000003 | 0.000004 |
| Candidatus_Halomonas_phosphatis | 0.000000 | 0.000000 | 0.000004 |
| Faecalicoccus_pleomorphus | 0.000001 | 0.000002 | 0.000006 |
| Clostridium_sp_CL-6 | 0.000001 | 0.000001 | 0.000004 |
| Pigmentiphaga_daeguensis | 0.000004 | 0.000000 | 0.000000 |
| Eubacterium_sp_1-3 | 0.000004 | 0.000000 | 0.000000 |
| Succinatimonas_hippei | 0.000000 | 0.000004 | 0.000000 |
| Nitrosomonas_europaea | 0.000000 | 0.000000 | 0.000004 |
| Clostridium_sp_Culture-27 | 0.000004 | 0.000005 | 0.000000 |
| Clostridium_sp_YIT_12069 | 0.000007 | 0.000002 | 0.000005 |
| Pantoea_sp_PSNIH2 | 0.000007 | 0.000003 | 0.000003 |
| Weissella_cibaria | 0.000002 | 0.000004 | 0.000002 |
| Mailhella_massiliensis | 0.000002 | 0.000012 | 0.000006 |

| Cyanobacteria/Melainabacteria_group_bacterium_S15 |  | | |
| --- | --- | --- | --- |
| B-MN24_CBMW_12 | 0.000003 | 0.000002 | 0.000000 |
| Paraprevotella_xylaniphila | 0.000003 | 0.000003 | 0.000000 |
| Prevotella_histicola | 0.000003 | 0.000000 | 0.000000 |
| Fusobacterium_mortiferum | 0.000000 | 0.000003 | 0.000000 |
| Actinomadura_keratinilytica | 0.000003 | 0.000000 | 0.000000 |
| Bittarella_massiliensis | 0.000004 | 0.000000 | 0.000003 |
| Lactobacillus_fermentum | 0.000000 | 0.000003 | 0.000000 |
| Nitrospira_sp | 0.000000 | 0.000000 | 0.000003 |
| Sporosarcina_globispora | 0.000000 | 0.000003 | 0.000000 |
| Ruminococcus_flavefaciens | 0.000000 | 0.000003 | 0.000000 |
| Sutterella_sp_KLE1602 | 0.000003 | 0.000000 | 0.000001 |
| Paenibacillus_montaniterrae | 0.000003 | 0.000000 | 0.000000 |
| Lachnospiraceae_bacterium_DW67 | 0.000003 | 0.000000 | 0.000000 |
| Moraxella_atlantae | 0.000002 | 0.000000 | 0.000000 |
| Vampirovibrio_chlorellavorus | 0.000000 | 0.000002 | 0.000000 |
| Gemmatimonas_sp_WX54 | 0.000000 | 0.000002 | 0.000000 |
| Bdellovibrio_sp_SKB1291214 | 0.000000 | 0.000000 | 0.000002 |
| Bacillus_thermoamylovorans | 0.000002 | 0.000000 | 0.000000 |
| Oligoflexus_tunisiensis | 0.000002 | 0.000000 | 0.000000 |
| Actinomyces_hyovaginalis | 0.000002 | 0.000000 | 0.000000 |
| Roseomonas_lacus | 0.000002 | 0.000000 | 0.000001 |
| Acidobacteria_bacterium_SCN_69-37 | 0.000000 | 0.000000 | 0.000002 |
| Parapedobacter_soli | 0.000000 | 0.000002 | 0.000000 |
| Arcobacter_cryaerophilus | 0.000000 | 0.000002 | 0.000000 |
| Bacillaceae_bacterium_NS1-3 | 0.000002 | 0.000000 | 0.000000 |
| Actinomyces_graevenitzii | 0.000000 | 0.000000 | 0.000002 |
| Gluconobacter_cerinus candidate_division_WOR- | 0.000001 | 0.000002 | 0.000001 |
| 1_bacterium_RIFCSPHIGHO2_01_FULL_53_15 | 0.000000 | 0.000001 | 0.000000 |
| Clostridium_sp_AL05-12 | 0.000000 | 0.000000 | 0.000001 |
| Ruthenibacterium_lactatiformans | 0.000000 | 0.000001 | 0.000002 |
| Terrimonas_sp_LX75 | 0.000000 | 0.000001 | 0.000000 |
| Flavobacteria_bacterium_BAL38 | 0.000000 | 0.000001 | 0.000000 |
| [Clostridium]_leptum | 0.000001 | 0.000000 | 0.000000 |
| Peptoniphilus_sp_EL1 | 0.000001 | 0.000000 | 0.000001 |
| Streptococcus_sp_feline_oral_taxon_345 | 0.000001 | 0.000000 | 0.000000 |
| Rhodococcus_erythropolis | 0.000001 | 0.000001 | 0.000000 |
| Others | 0.513010 | 0.449095 | 0.526091 |

Supplementary Figure 1. Effective Tags data, low-frequency Tags data and Tags annotation data of all samples. Generated by using R Software^1^


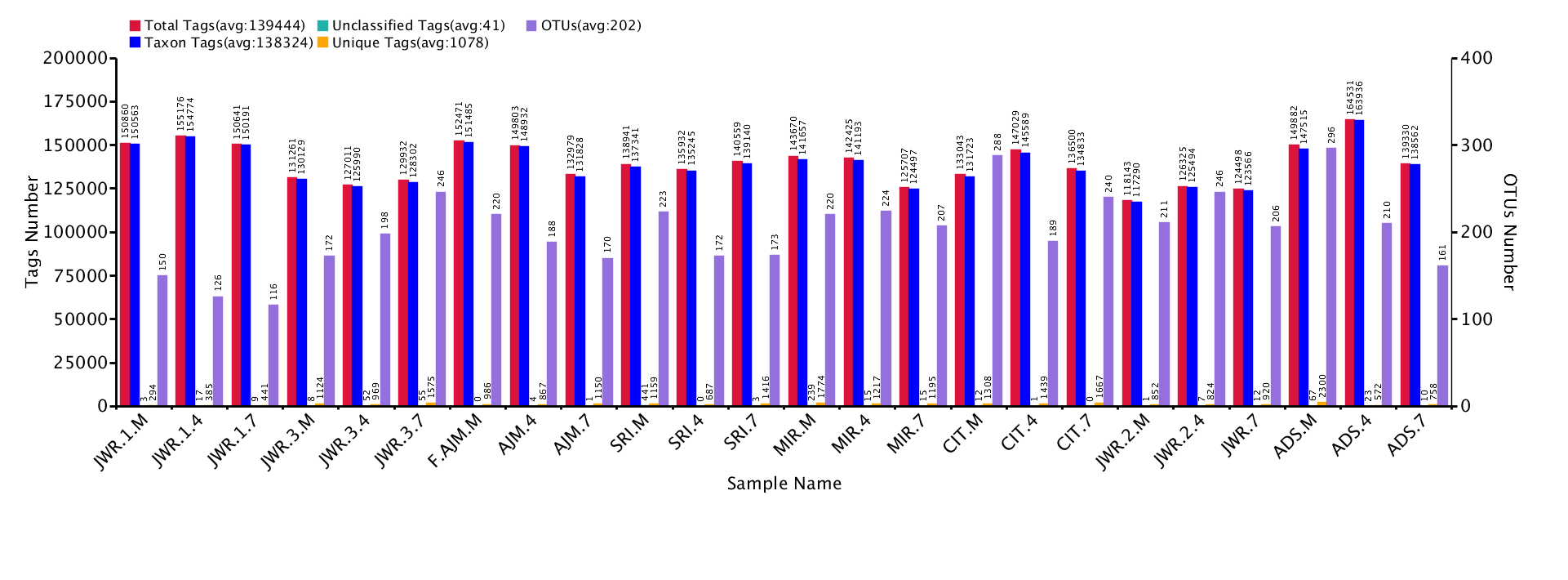


Supplementary Figure 2. Taxonomic annotation of all subjects at three-time point

Generated by using R Software^1^


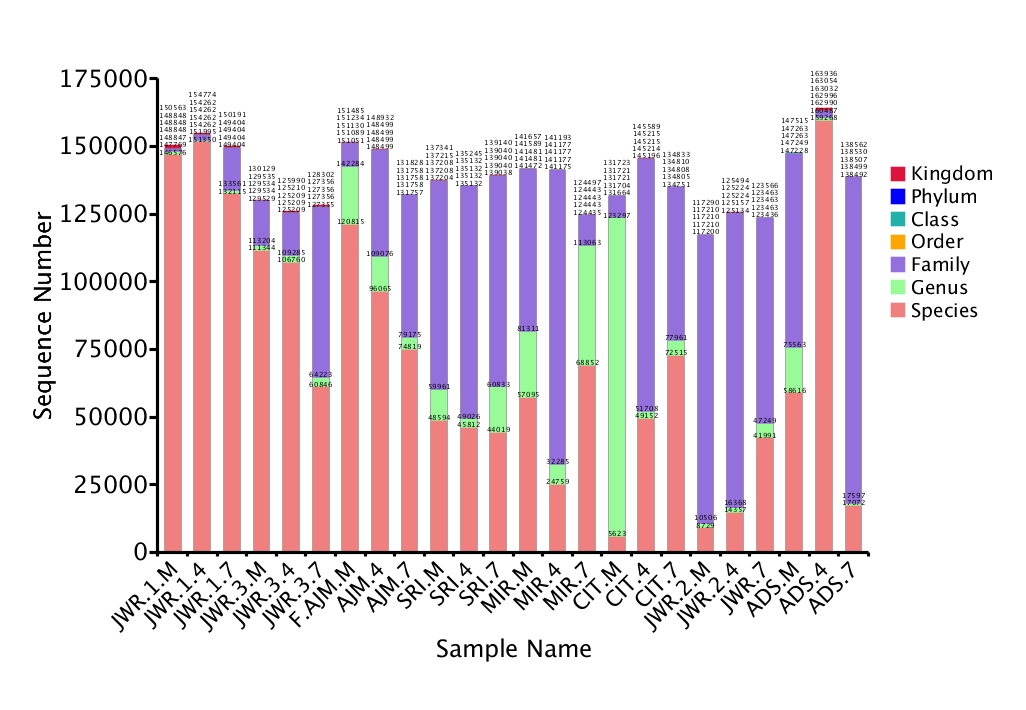


Supplementary Figure 3 Taxonomic annotation in the phylum level of all subjects at three-time points

Generated by using R Software^1^


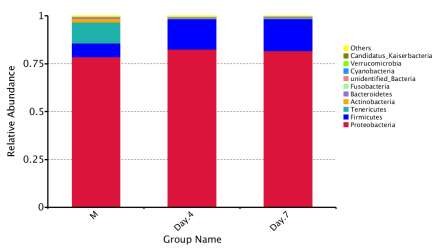


Supplementary Figure 4 Taxonomic annotation in the class level of all subjects at three-time points

Generated by using R Software^1^


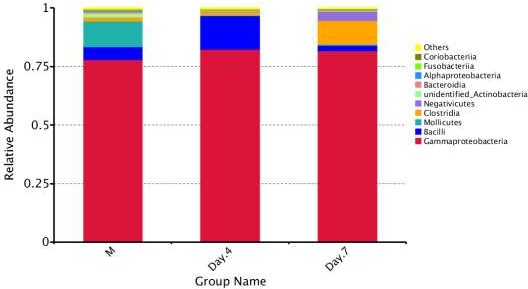


Supplementary Figure 5 Taxonomic annotation in the order level of all subjects at three-time points

Generated by using R Software^1^


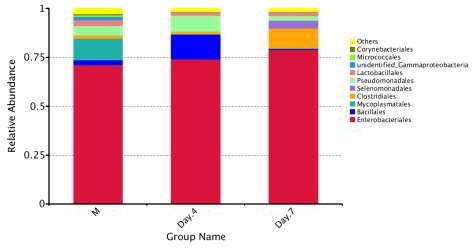


Supplementary Figure 6 Taxonomic annotation in the family level of all subjects at three-time points

Generated by using R Software^1^


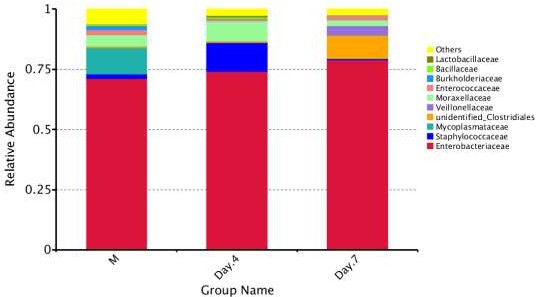


Supplementary Figure 7. Taxonomic annotation in the genus level of all subjects at three-time points

Generated by using R Software^1^


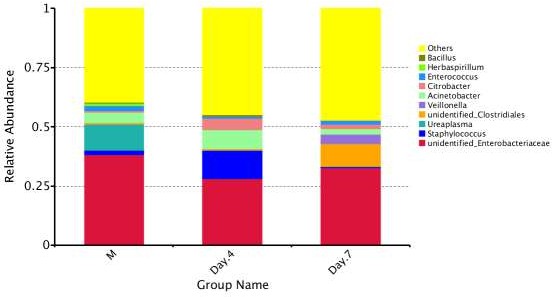


Supplementary Figure 8. Phylogenetic tree visualization of taxonomic annotation in the kingdom until species level in meconium. Different colours represent different taxonomic ranks. The size of circles represents the relative abundance of species. The first number below the taxonomic name represents the percentage in the whole taxon, while the second number represents the percentage in the selected taxon.

Generated by using R Software^1^


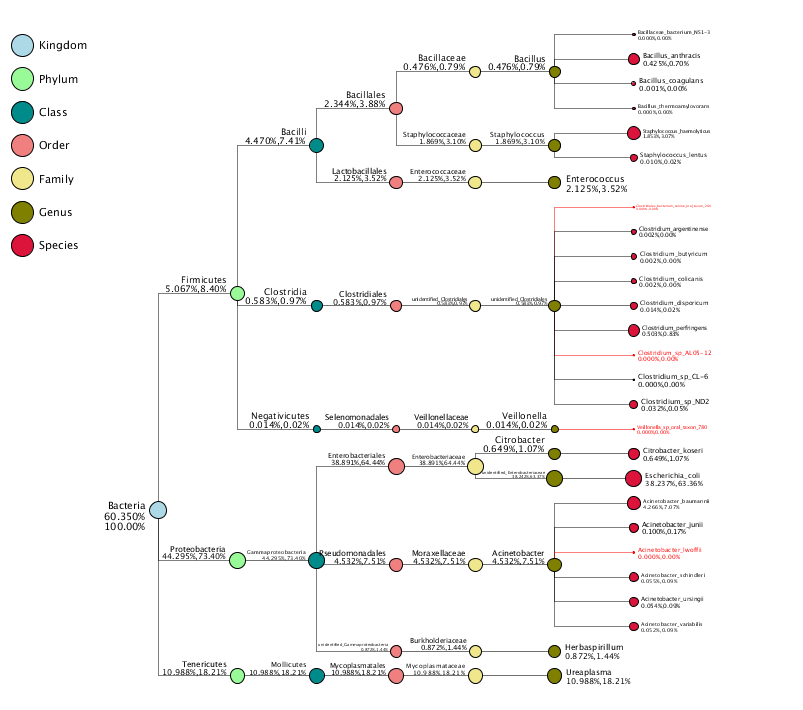


Supplementary Figure 9. Phylogenetic tree visualization of taxonomic annotation in the kingdom until species level in feces at day 4. Different colours represent different taxonomic ranks. The size of circles represents the relative abundance of species. The first number below the taxonomic name represents the percentage in the whole taxon, while the second number represents the percentage in the selected taxon.

Generated by using R Software^1^


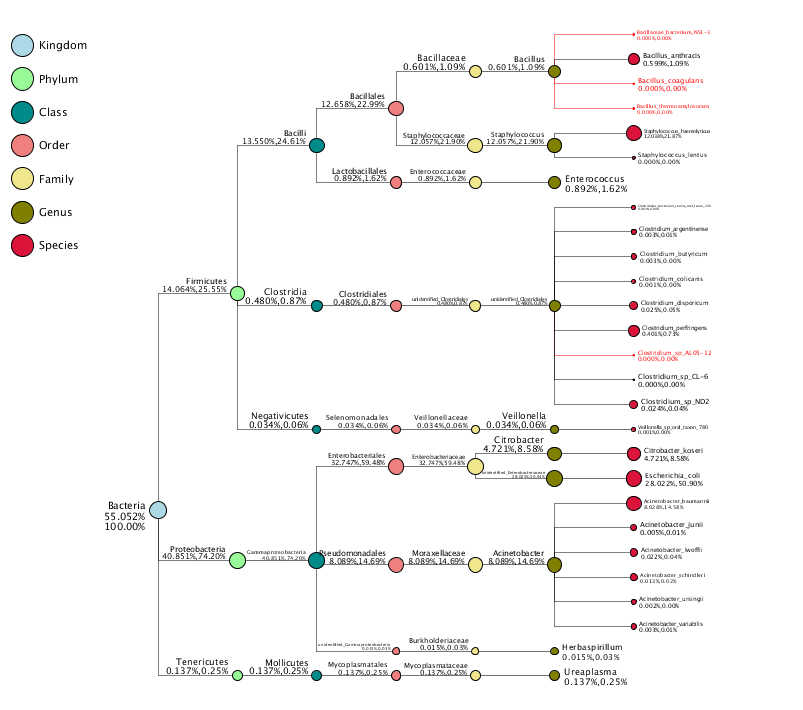


Supplementary Figure 10. Phylogenetic tree visualization of taxonomic annotation in the kingdom until species level in feces at day 7. Different colours represent different taxonomic ranks. The size of circles represents the relative abundance of species. The first number below the taxonomic name represents the percentage in the whole taxon, while the second number represents the percentage in the selected taxon.

Generated by using R Software^1^


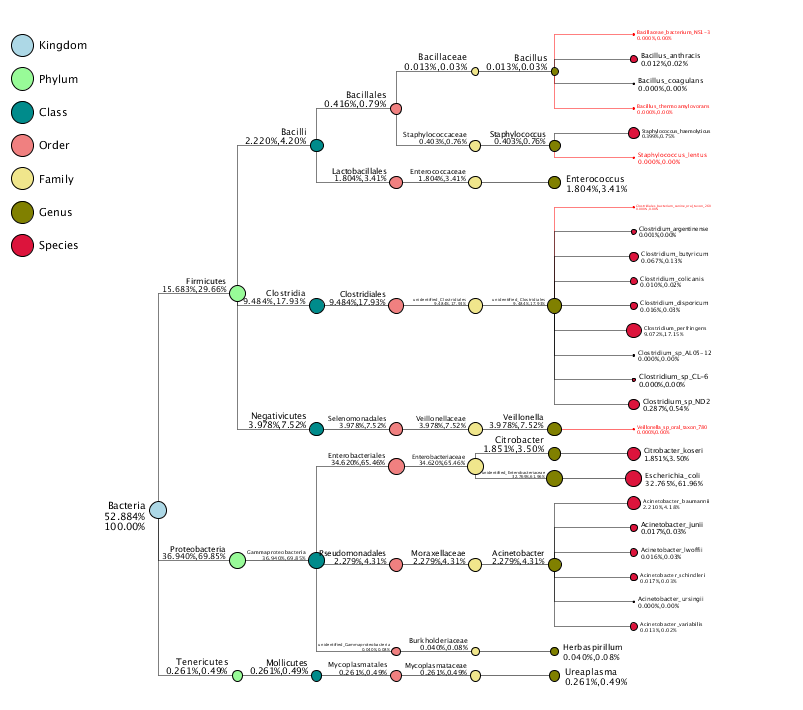


Supplementary Figure 11. Rarefaction measures analysis by chao1, observed species, Shannon and PD to indicate whether the biodiversity of microbiome samples reflects the rationality of the sequencing data volume directly and reflects the richness of microbial community in the samples indirectly showing flatter curves.

Generated by using R Software^1^


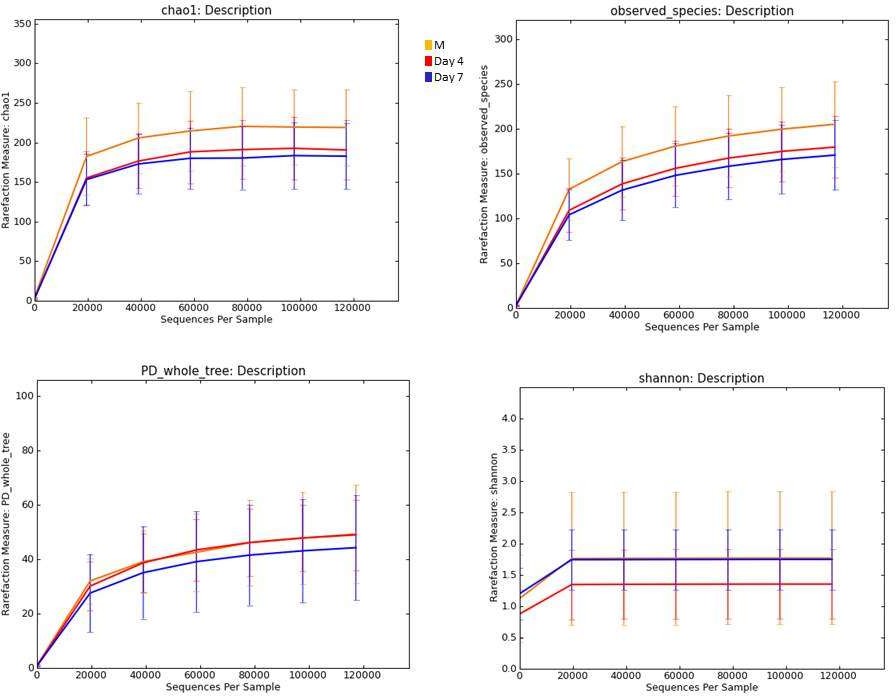


Supplementary Figure 12. Cluster tree construction of UPGMA based on the Weighted Unifrac Distance considering high-abundance taxa to achieve the underlying driving factors to determine the complexity differences of microbiome in meconium, feces Day 4 and feces Day 7

Generated by using R Software^1^


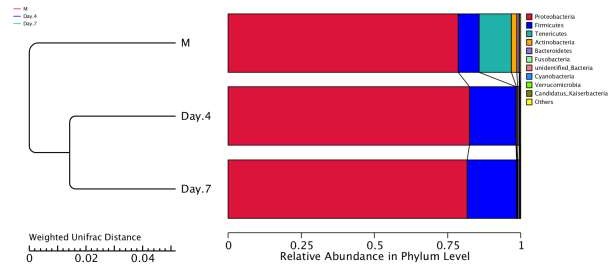


Supplementary Figure 13. Cluster tree of UPGMA based on the Unweighted Unifrac Distance considering high-abundance taxa to achieve the underlying driving factors to determine the complexity differences of microbiome in meconium, feces Day 4 and feces Day 7

Generated by using R Software^1^


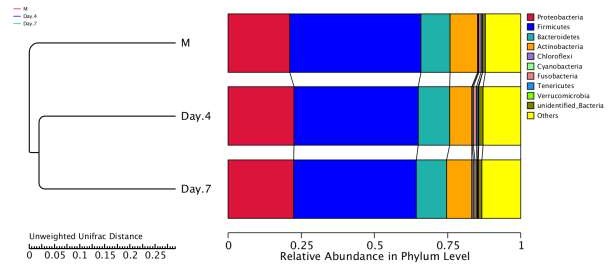


Supplementary Figure 14. The changes and development of microbiota diversity and proportion of triplets JWR1, JWR2 and JWR3 stools at three time-points, i.e. JWR1.M, JWR1.Day4, JWR1.Day7, JWR2.M, JWR2.Day4, JWR2.Day7, JWR3.M, JWR3.Day4 and JWR3.Day7 by Krona displays. The most abundant Phylum Proteobacteria and its most abundant Genus Enterobacteriaceae, and the second most abundant Phylum Firmicutes

Generated by using R Software^1^


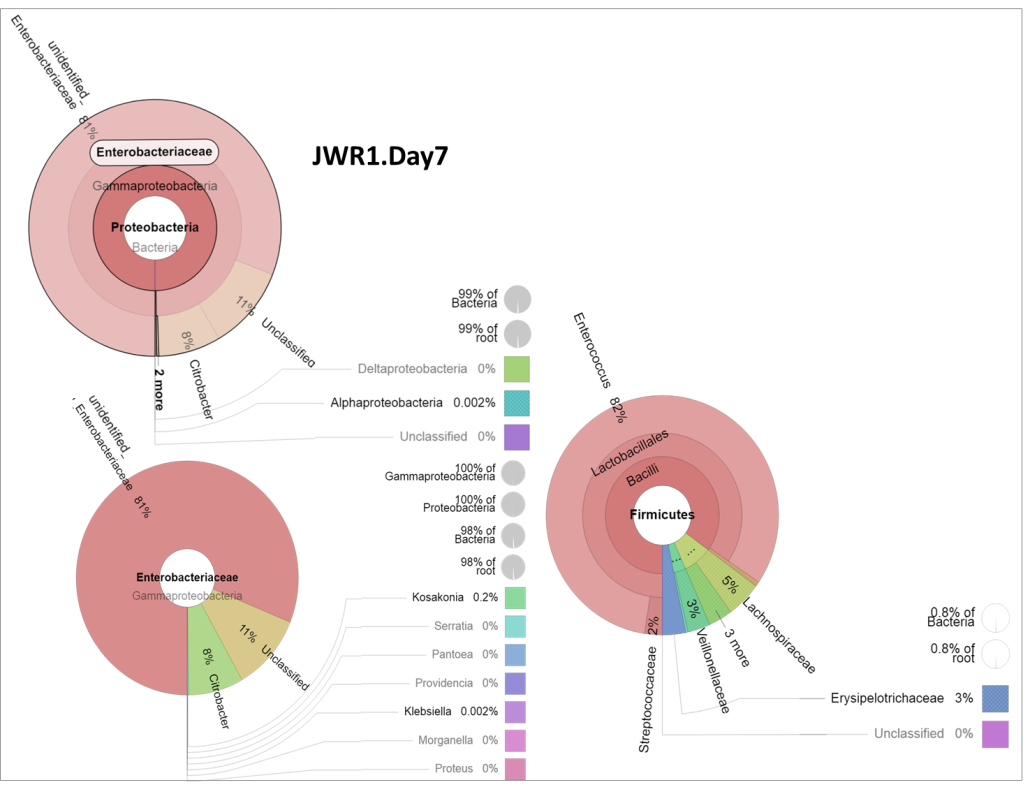

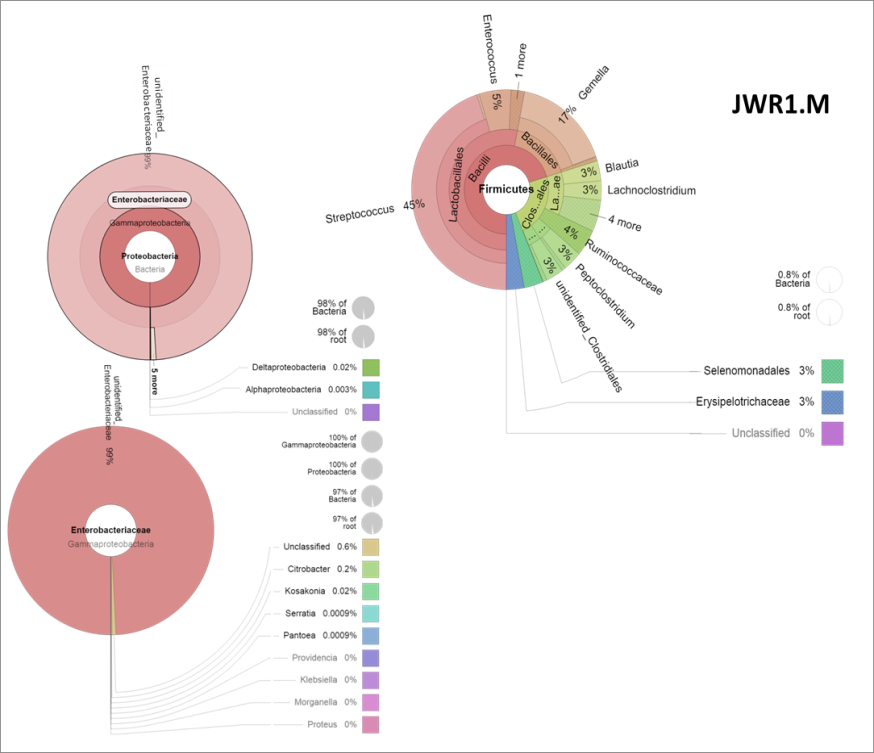

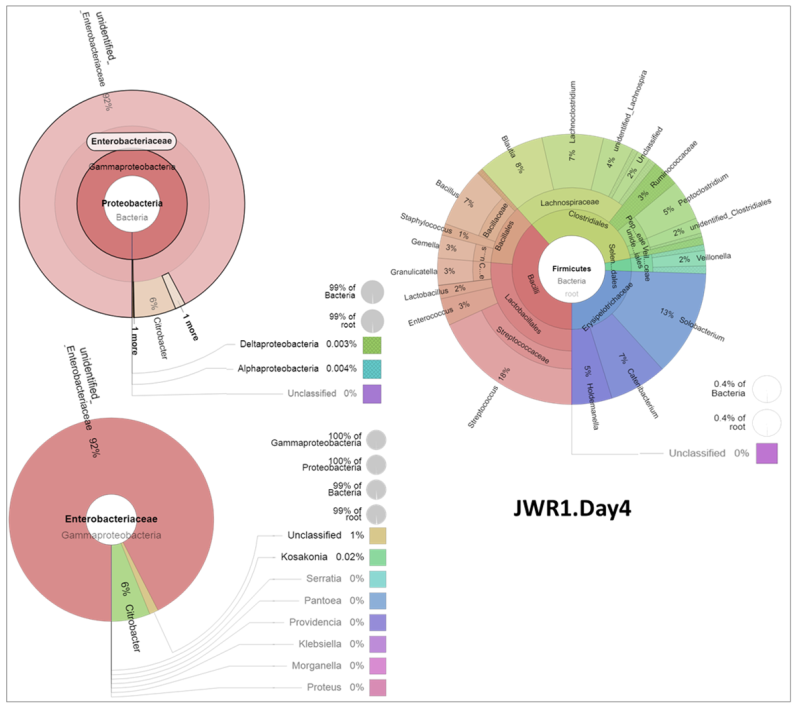


The changes and development of microbiota diversity and proportion of triplets JWR1, JWR2 and JWR3 stools at three time-points, i.e. JWR1.M, JWR1.Day4, JWR1.Day7, JWR2.M, JWR2.Day4, JWR2.Day7, JWR3.M, JWR3.Day4 and JWR3.Day7 by Krona displays. The most abundant Phylum Proteobacteria and its most abundant Genus Enterobacteriaceae, and the second most abundant Phylum Firmicutes


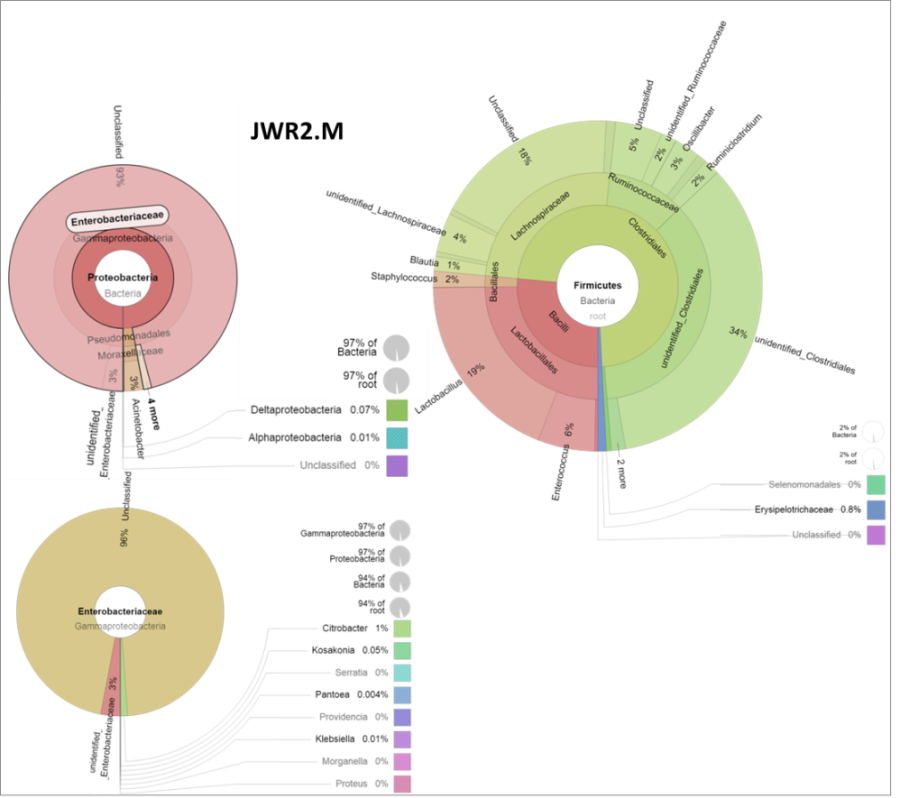

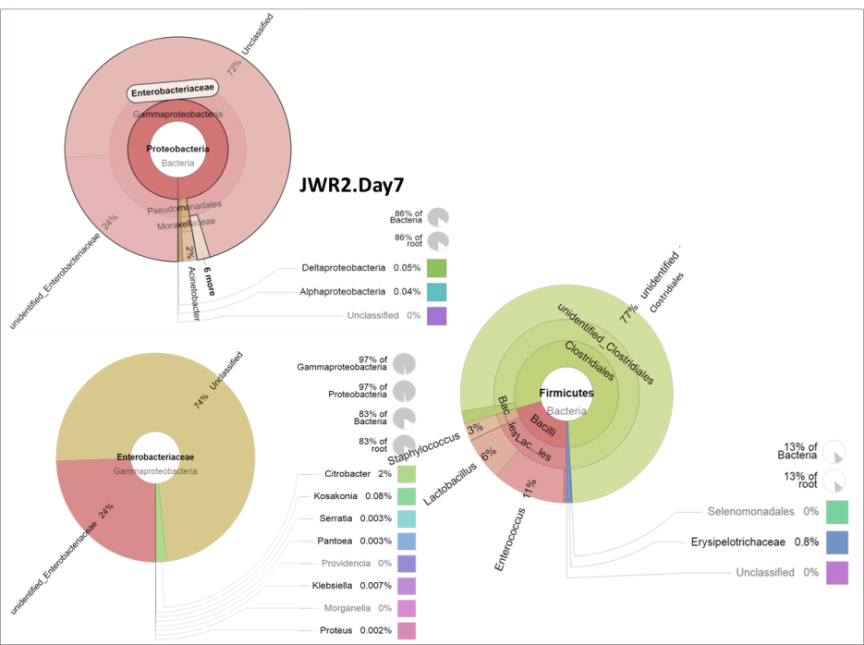

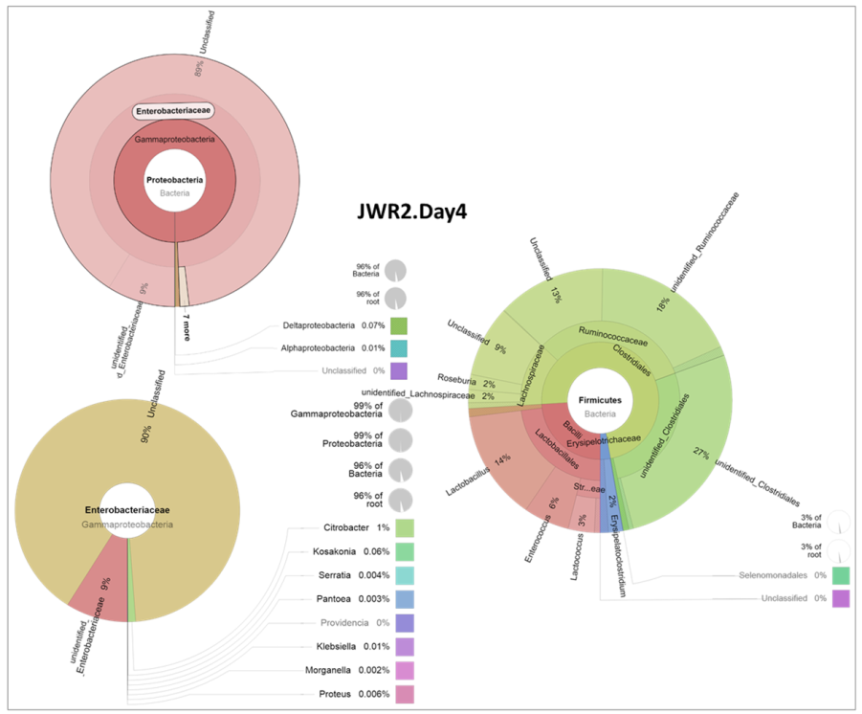


The changes and development of microbiota diversity and proportion of triplets JWR1, JWR2 and JWR3 stools at three time-points, i.e. JWR1.M, JWR1.Day4, JWR1.Day7, JWR2.M, JWR2.Day4, JWR2.Day7, JWR3.M, JWR3.Day4 and JWR3.Day7 by Krona displays. The most abundant Phylum Proteobacteria and its most abundant Genus Enterobacteriaceae, and the second most abundant Phylum Firmicutes


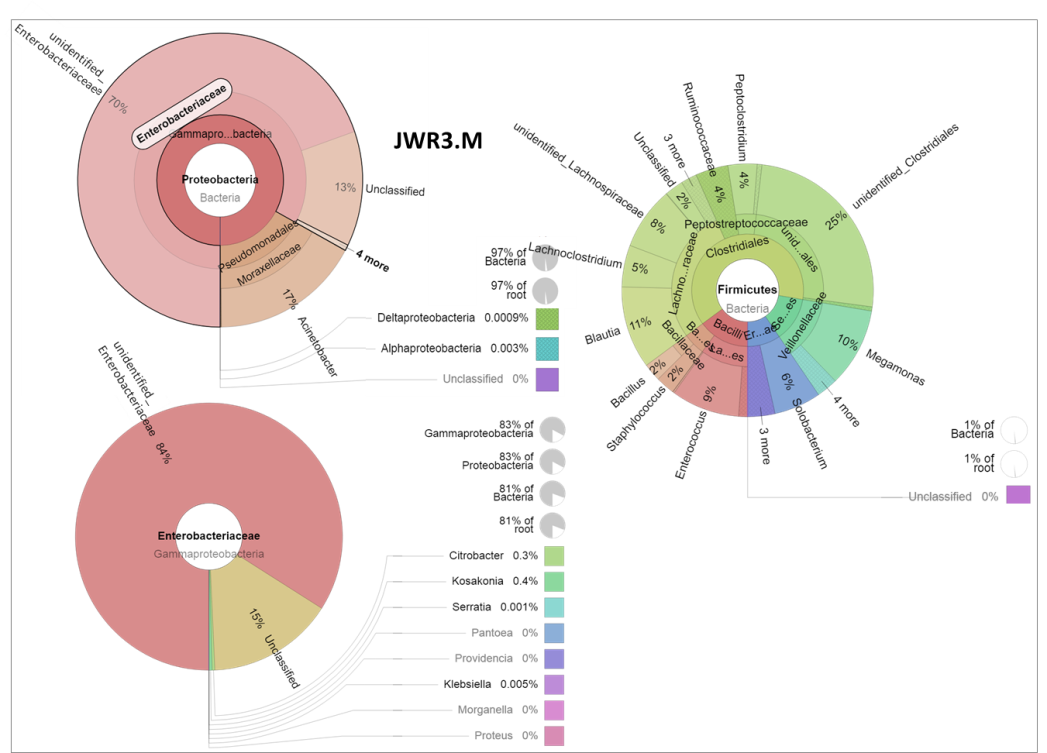

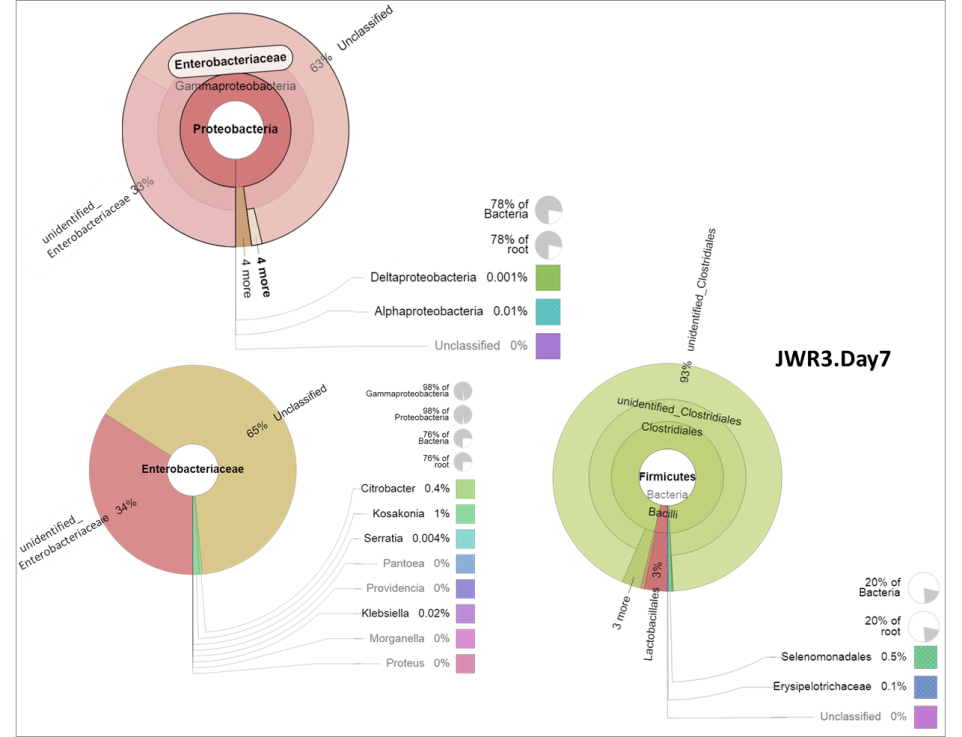

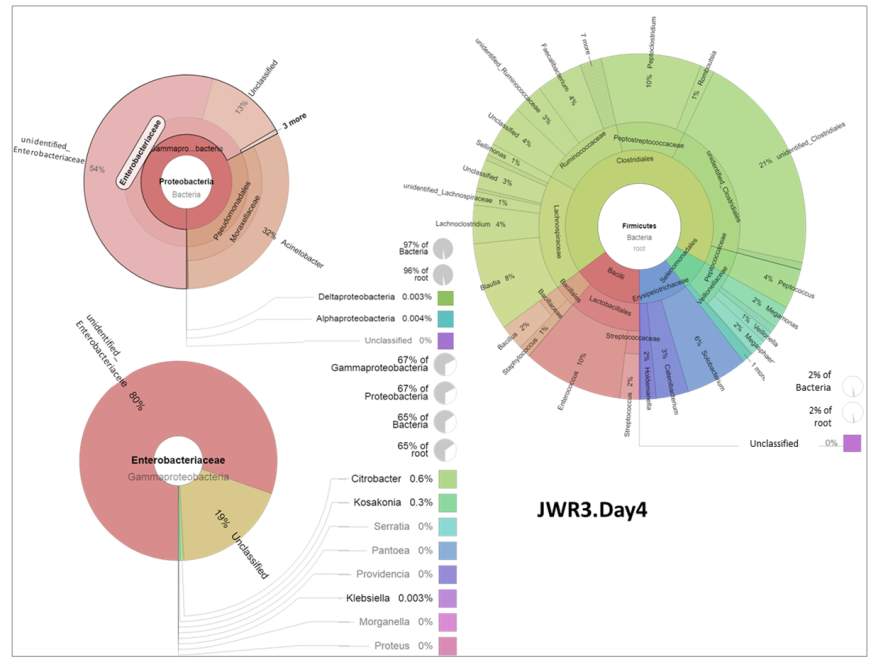


Supplementary Figure 15. Venn diagram representing the diversity of microbiome at three-time points groups of triplets JWR1, JWR2, and JWR3.

Generated by using R Software^1^


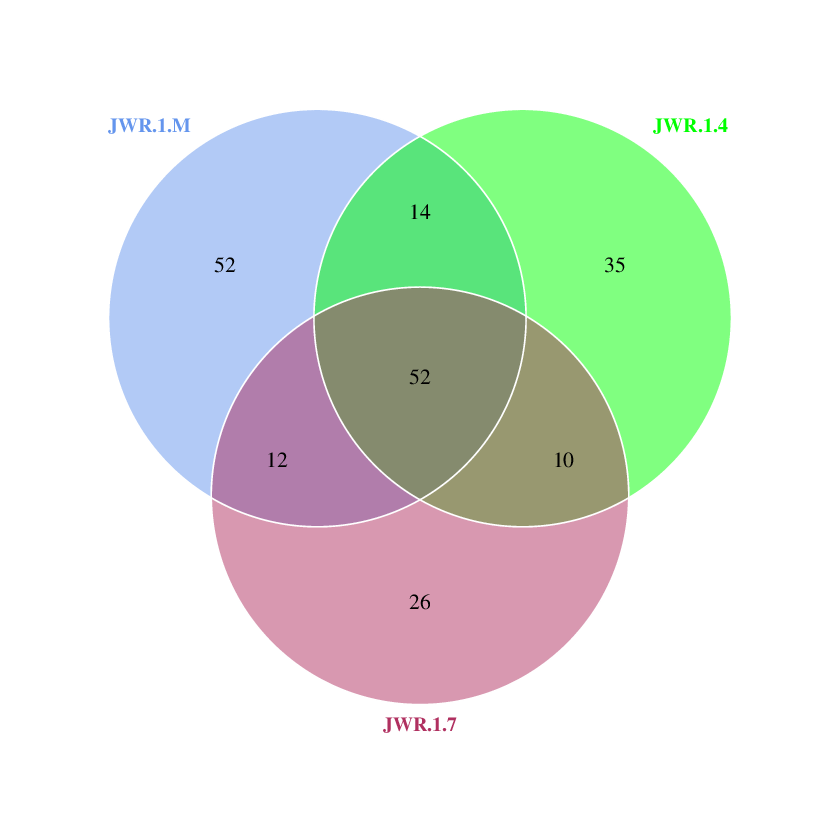

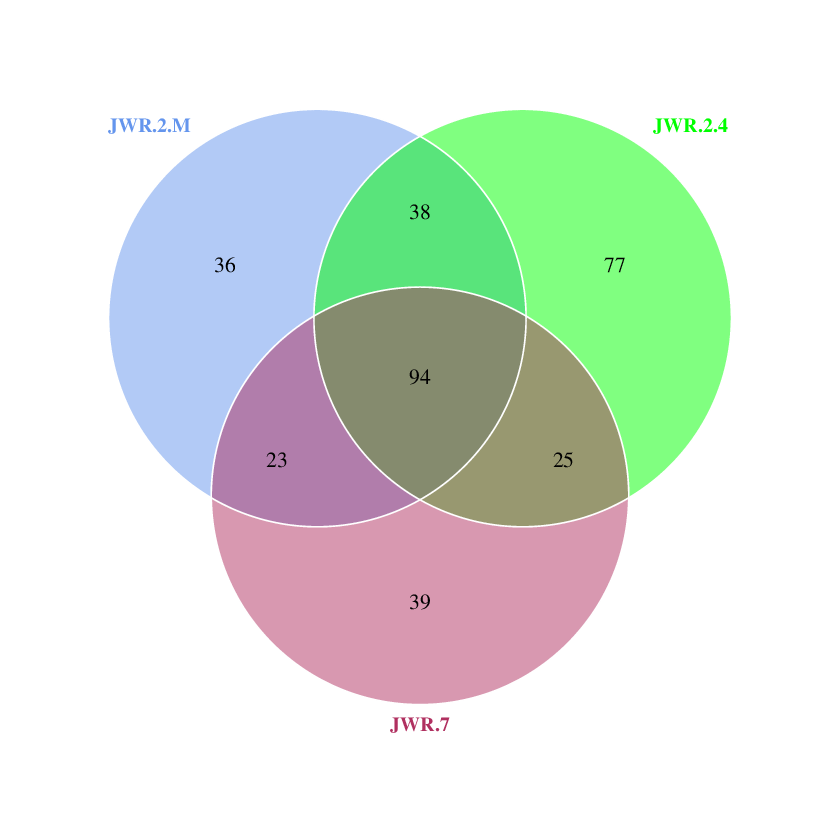


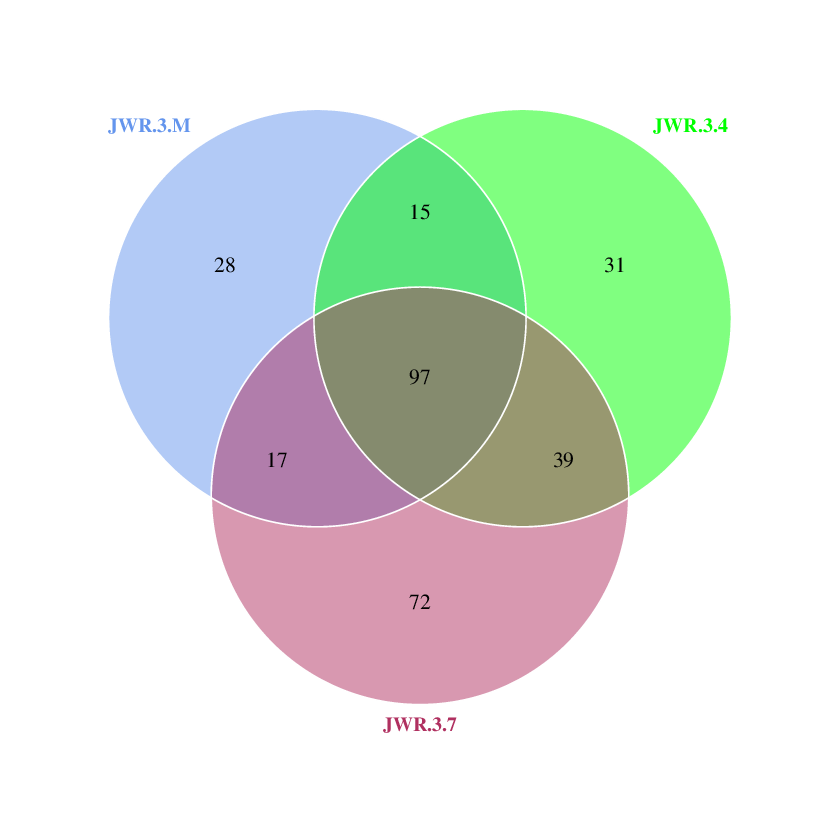

Supplement: Supplementary file 1 — Supplementary Information. [file 41598_2022_13496_MOESM1_ESM.docx]
